# Isolation and characterization of broadly-neutralizing anti-HCMV-gB antibodies from human donors using a prefusion-stabilized HCMV gB variant

**DOI:** 10.1371/journal.ppat.1013950

**Published:** 2026-02-05

**Authors:** Maria K. McClave, Yu-Hsin Wan, Ellen M. White, Beatriz Gálvez Martínez, Mohammad Karimian Shamsabadi, Nicholas Aldridge, Bibhav Poudel, Adrian W. Sperl, Andrew T. McGuire, Ekaterina E. Heldwein

**Affiliations:** 1 Department of Molecular Biology and Microbiology, Tufts University School of Medicine, Boston, Massachusetts, United States of America; 2 Graduate Program in Molecular Microbiology, Graduate School of Biomedical Sciences, Tufts University School of Medicine, Boston, Massachusetts, United States of America; 3 Vaccine and Infectious Disease Division, Fred Hutchinson Cancer Center, Seattle, Washington, United States of America; 4 Faculty of Experimental Sciences, Department of Biotechnology, University of Francisco de Vitoria, Madrid, Spain; 5 Graduate Program in Genetics, Molecular, and Cell Biology, Graduate School of Biomedical Sciences, Tufts University School of Medicine, Boston, Massachusetts, United States of America; 6 Department of Global Health, University of Washington, Seattle, Washington, United States of America; 7 Department of Laboratory Medicine and Pathology, University of Washington, Seattle, Washington, United States of America; Leibniz Institute of Virology (LIV), GERMANY

## Abstract

Human cytomegalovirus (HCMV) poses a significant risk to immunocompromised individuals and is the leading cause of congenital birth defects worldwide. There is no cure or robust treatment options, although neutralizing antibodies (nAbs) derived from patient sera are being explored as prophylactics with limited success. Glycoprotein B (gB) is a viral membrane fusogen and a major target of the anti-HCMV humoral response in humans. Here, we engineered a soluble, prefusion-stabilized HCMV gB ectodomain variant and used it to isolate twelve new human monoclonal antibodies (mAbs). Seven of these mAbs strongly neutralized at least one strain of HCMV *in vitro,* whereas six mAbs neutralized both lab-adapted and minimally passaged clinical strains (were broadly neutralizing, bnAbs). All nAbs bound different epitopes within antigenic regions AD-4 or AD-5, and most targeted new sites. Despite being isolated using prefusion-stabilized HCMV gB variant, nAbs varied in their conformational specificity. Only one nAb preferentially bound the prefusion form, and most preferentially bound the intermediate form. The seven nAbs were separated into three classes based on their putative neutralization mechanisms, which were deduced from their conformational specificity, reactivity with gB on the cell surface, and epitope location. Our stabilized prefusion-gB construct provides a tool for isolating potent new nAbs, including prefusion-specific ones, and studying HCMV immunogenicity. Long term, these potent nAbs that arose during natural infections could be developed into potent prophylactics and therapeutics against HCMV diseases.

## Author summary

Human cytomegalovirus (HCMV) infects the majority of people worldwide but poses the greatest risk to immunocompromised individuals. There is no cure, vaccine, or effective treatment option for HCMV, but human polyclonal antibodies have been tested with limited success. To infect cells, HCMV uses the viral membrane fusogen glycoprotein B (gB), which exists in metastable prefusion, stable postfusion, and highly dynamic intermediate conformations. We engineered a soluble, prefusion-stabilized HCMV gB ectodomain construct and, in the process, also designed the first intermediate HCMV gB construct. We used our prefusion gB construct to isolate twelve new human monoclonal antibodies (mAbs), six of which were broadly neutralizing against multiple HCMV strains. Interestingly, only one mAb preferentially bound the prefusion form while most favored the intermediate form, and this did not correlate with their neutralization potency. We further characterized these mAbs using a variety of biochemical, cell-based, and low-resolution structural approaches and identified their putative neutralization mechanisms. Using our methodology, we also identified neutralization mechanisms for two previously published nAbs. Our constructs introduce new tools into the field of HCMV, and, in the long term, the nAbs isolated in this study could aid in the development of robust HCMV countermeasures.

## Introduction

Human cytomegalovirus (HCMV) is a beta-herpesvirus that has been associated with significant morbidity since its first description over a century ago [reviewed in [1]]. Despite eliciting both humoral and cell-mediated immune responses [reviewed in [2,3]], HCMV establishes a lifelong infection in more than 90% of the population, with latency in hematopoietic progenitor cells [reviewed in [2–4]]. While infection is typically asymptomatic in immunocompetent individuals, HCMV poses a significant risk to immunocompromised individuals and congenitally infected infants [Reviewed in [5,6]], leading to developmental impairments in the latter group [reviewed in [4]]. Unfortunately, no vaccines, prophylactics, or safe therapeutics exist to counteract the significant morbidity of HCMV.

Unlike most viruses that rely on a single glycoprotein for entry into target host cells, HCMV uses complex multi-protein machinery [reviewed in [7–10]]. Three glycoproteins, conserved across all herpesviruses – gB, gH, and gL – are essential and sufficient for membrane fusion [11]. A hyper-fusogenic chimera of gB can mediate cell-cell fusion alone, highlighting the importance of this protein in target cell entry [12]. Entry into all cell types requires initial tethering to heparan sulfate [13]. Entry into epithelial or endothelial cells additionally requires the HCMV-specific pentamer (gH/gL/UL128/UL130/UL131) [14,15], which binds cellular receptors such as Nrp-2 [16] and OR14I1 [17]. A new gH/UL116/UL141 complex enhances entry into endothelial cells [18]. Entry into fibroblasts requires the HCMV-specific trimer (gH/gL/gO), which binds platelet-derived growth factor receptor alpha [19–21]. The trimer is additionally required for entry into all cell types, including fibroblasts, epithelial, endothelial, and myeloid cells [22].

**Fig 1:**
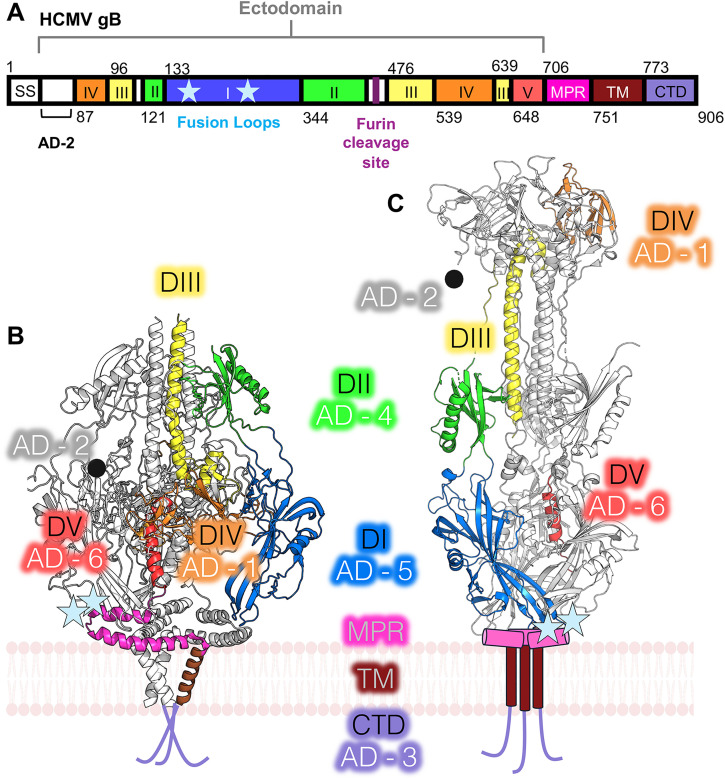
HCMV gB is composed of five structural and antigenic domains. **(A)** Domain map of full-length HCMV gB. Division of domains indicated by color and residue number. Unlabeled white boxes indicate unresolved regions. The membrane-proximal region (MPR) is shown in magenta. The transmembrane region (TM) is shown in brown. The cytoplasmic domain (CTD) is shown in light purple. Fusion loop sites are shown as light blue stars in dI and labeled accordingly. The furin cleavage site is represented by a dark purple line between dII and dIII and is labeled accordingly. **(B)** Ribbon model of prefusion HCMV gB with one monomer colored by the domain (*RCSB 7KDP*) with structural and antigenic domains (AD) indicated. Fusion loop locations are marked with light blue stars. Light purple lines represent the CTD. **(C)** Ribbon model of postfusion HCMV gB with one monomer colored by domain (*RCSB 5CXF*). MPR and TM are represented by magenta and brown cylinders, respectively. Fusion loop locations are marked with light blue stars. Light purple lines represent the CTD. Each structural domain with its corresponding antigenic domain (AD) is indicated.

gB is the conserved fusogen of herpesviruses. Fusogens are viral surface glycoproteins that mediate the merger of the viral envelope and host membrane during entry and cell spread in all enveloped viruses by refolding from the metastable prefusion to the stable postfusion form [23]. The high-resolution structures of both the prefusion [24] and postfusion forms [25,26] of the HCMV gB ectodomain have been determined, providing key insights into the structural transitions required for membrane fusion (Fig 1). In both forms, gB is a trimer, with each protomer organized into five structural domains (dI-V), which undergo distinct rearrangements during fusion (Fig 1). Domain I (dI) consists of a pleckstrin homology domain (PHD) module and finger-like β-sheet protrusions that terminate in fusion loops (Fig 1, blue). This domain remains largely unchanged between the two conformations, retaining its overall structure and membrane-proximal position (Fig 1B and 1C, blue). DII consists of another PHD and, as dI, largely retains its conformation during the pre- to postfusion transition (Fig 1B and 1C, green). dIII has a long α-helix that assembles into a central triple coiled-coil within the gB trimer (Fig 1, yellow). This coiled-coil extends upward during fusion, forming the support base for dIV in the postfusion conformation (Fig 1B and 1C, yellow). In the postfusion conformation, dIV forms ear-like extensions at the distal end of gB, opposite the fusion loops (Fig 1, orange). Similar to dIII, it extends upward from its buried position within the prefusion conformation to form the top of the postfusion structure (Fig 1B and 1C, orange). Lastly, dV is an elongated polypeptide that runs nearly the entire length of postfusion gB, fitting into the groove formed by dIII and dI of two adjacent protomers (Fig 1, red). It remains primarily buried in the pre- and postfusion forms (Fig 1B and 1C, red).

HCMV infection elicits both humoral and cell-mediated immune responses [2,3,27,28], with most neutralizing antibodies (nAbs) – Abs that interfere with the ability of the virus to enter a cell *in vitro* – targeting HCMV gH/gL [29]. In addition to gH/gL, a significant portion of known nAbs target gB, establishing gB as a major target of the anti-HCMV humoral response [30]. Thus far, six antigenic regions within gB, termed antigenic domains (AD), have been defined (Fig 1C). Antigenic domain 1 (AD-1), located within dIV, was the first AD to be characterized based on some of the first anti-gB nAbs binding this region [31]. AD-2 is situated in the first 80 N-terminal residues [32,33], which are unresolved in the known structures [24–26]. This is the only antigenic domain known to have two distinct antigenically dominant sites [33]. The first, Site I, lies between residues 68 and 77. The second, Site II, lies between residues 50 and 54. AD-3 is, surprisingly, located within the cytoplasmic domain of gB [34]. Finally, AD-4, AD-5, and AD-6 are located within dII, dI, and dV respectively [30,35].

gB-based vaccines, such as an MF59-adjuvanted gB subunit vaccine, have shown partial protection (ability to prevent morbidity and mortality *in vivo*) [28,36]. However, this protection was thought to be mediated by non-neutralizing mechanisms such as antibody-dependent cytotoxicity (ADCC) and antibody-dependent cellular phagocytosis (ADCP) [37,38], instead of nAb responses, which are important correlates of protection for successful viral vaccines [[28,38,39]; reviewed in [40,41]]. A gB-based vaccine eliciting strong nAb responses could offer robust protection. Indeed, a new vaccine candidate currently in late-stage efficacy trials, mRNA-1647, combines gB and the HCMV-specific pentamer complex to elicit robust nAbs [42].

Although mAb therapies are being explored, the current standard of care in some regions remains patient-derived polyclonal immunoglobulin, which has limited efficacy and poses standardization and safety concerns [5,28,43,44]. For this reason, it is not widely used in the US [44]. Studies in other viruses, such as respiratory syncytial virus (RSV), have demonstrated that Abs targeting prefusion conformations of fusion proteins are highly potent neutralizers [39,45,46]. Thus, identifying prefusion-gB-specific nAbs could lead to a well-defined HCMV mAb therapy.

A major obstacle in isolating prefusion-gB-specific nAbs has been stabilizing the prefusion form of gB. When soluble gB ectodomain is expressed, it spontaneously adopts the postfusion form due to the metastable nature of the prefusion form [12,25]. As a result, previous studies that analyzed Ab responses to HCMV gB used the soluble, postfusion form of gB [12,26,30,47,48]. But while some Abs raised against the postfusion form are nAbs, they are not protective [38]. All known gB-specific nAbs can bind to the postfusion form [26,30]. Although the postfusion form is highly immunogenic, it is not actively involved in the fusion process. This could explain why nAbs that can bind the postfusion form may be suboptimal in their neutralization and protection properties. By contrast, prefusion-gB-specific nAbs would be predicted to be both strongly neutralizing and protective. However, no prefusion-gB-specific anti-HCMV Abs have yet been identified or characterized. Thus, there is an urgent need to devise a strategy to stabilize gB in its prefusion conformation, enabling the isolation of prefusion-specific nAbs.

To address this need, we engineered a soluble, prefusion-stabilized HCMV gB ectodomain variant and utilized it to isolate 12 new human mAbs, seven of which neutralize at least one HCMV strain. While some of the seven new nAbs competed with known nAbs, most targeted new epitopes within dI (AD-5) and dII (AD-4). Most neutralizing antibodies bound the prefusion and intermediate forms. Notably, one potent nAb preferentially bound the prefusion form, underscoring the importance of conformational preference. Our stabilized prefusion-gB variant provides a tool for isolating prefusion-specific nAbs and studying HCMV immunogenicity. Long term, this could enable the development of immunogens based on the stabilized prefusion form of HCMV gB – a strategy that has advanced vaccines based on RSV F protein [39,45].

## Results

### The design and validation of the soluble, prefusion-stabilized HCMV gB ectodomain

gB readily adopts the postfusion conformation when produced in its ectodomain form [25] or when extracted from the membrane [49]. Previously, full-length HCMV gB was stabilized in the prefusion form by a small-molecule ligand before detergent extraction from the viral envelope [24]. However, this method achieved only partial stabilization of the prefusion form, with only ~13% of gB adopting the prefusion conformation [24]. Here, we employed a protein engineering approach that leveraged available structural information to stabilize the prefusion form while destabilizing the postfusion form. A conceptually similar approach has been used with several viral fusogens [reviewed in [50]], including RSV F [51], SARS-CoV-2 Spike [52], and, more recently, HCMV gB [53]. The distinct architecture of the pre- and postfusion conformations (Fig 1) enabled the assessment of the conformational state of gB constructs by negative-stain electron microscopy (NS-EM). All constructs were initially produced in insect cells infected with recombinant baculoviruses.

**Fig 2:**
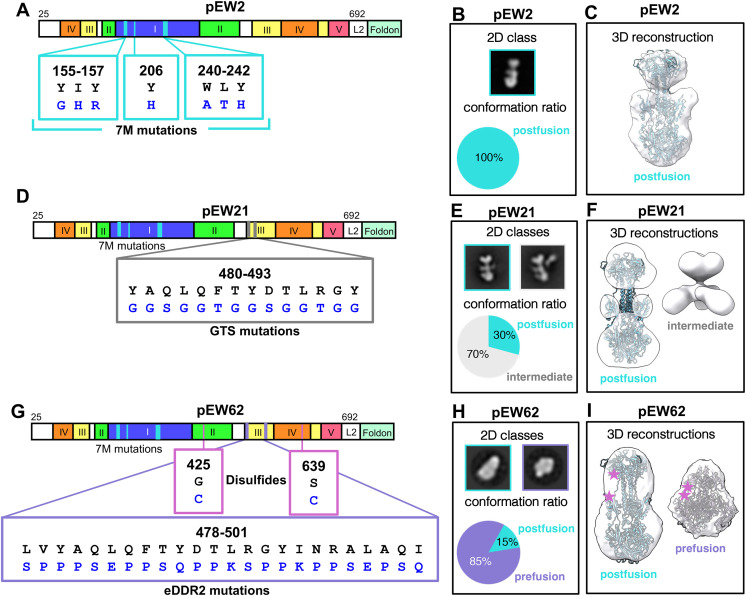
Design iterations to stabilize prefusion gB resulted in constructs representing three known gB conformations. **(A)** Domain map of construct pEW2 with mutation positions indicated. 7M mutations are indicated in cyan. **(B)** Negative stain electron microscopy (NS-EM) 2D class of conformations present in pEW2 sample. Conformation ratio as calculated by 2D classification particle counts with 12,489 particles total. **(C)** 3D reconstruction of pEW2 (1623 particles) overlaid with the structure of the known postfusion HCMV gB ectodomain (*RCSB: 5CXF*). **(D)** Domain map of construct pEW21 with mutation positions indicated. GTS mutations are indicated in gray. **(E)** Negative stain electron microscopy (NS-EM) 2D classes of conformations present in the pEW21 sample. (right) Conformation ratio as calculated by 3D classification particle counts. 5071 particles were recognizably postfusion, and 11,026 particles were deemed intermediate. **(F)** 3D reconstructions of pEW21 overlaid with the structure of the known postfusion HCMV gB ectodomain (*RCSB: 5CXF*) (5071 particles) or as an empty map (2929 particles). Additional intermediate 3D classes can be found in S1 Fig. **(G)** Domain map of construct pEW62 with mutation positions indicated. eDDR2 mutations are indicated in violet and disulfides in plum. **(H)** Negative stain electron microscopy (NS-EM) 2D classes (top) of conformations present in pEW62 sample. (Bottom) Conformation ratio as calculated by 2D classification particle counts. pEW62 contained 32,447 prefusion particles and 6,452 postfusion particles. **(I)** 3D reconstruction of conformations in pEW62 sample (prefusion = 8936 particles, postfusion = 2358 particles). Reconstructions are overlaid with known structures of prefusion HCMV gB ectodomain (*RCSB 7KDP*) and postfusion HCMV gB ectodomain (*RCSB 5CXF*). Locations of pEW62 disulfides are indicated with plum stars. Additional mutants made in attempts to stabilize prefusion gB can be found in S1 Table. Additional 2D classes of pEW2, pEW21, and pEW62, as well as additional 3D classes for pEW21 can be found in S1 Fig.

To begin, seven hydrophobic residues in the fusion loops were replaced with the corresponding hydrophilic residues from HSV-1 or EBV gB homologs to prevent the aggregation of the HCMV gB ectodomain [25]. These so-called “7M” mutations (Fig 2A) were included in all downstream constructs. Next, to stabilize the prefusion form, foldon, a trimerization domain from T4 phage fibritin [54], or GCN4t, a mutated leucine zipper from GCN4 that forms a trimeric coiled-coil [55], was added to the gB ectodomain C terminus (Fig 2A). The foldon and GCN4t trimers were expected to prevent the separation of the C termini necessary for the pre-to-post refolding [reviewed in [23]], thereby stabilizing the prefusion form. However, while successful at stabilizing the prefusion form of many fusogens [56–59], the trimerization tag approach failed to yield any form other than the postfusion (S1 Table). Therefore, one construct in this series, “pEW2”, was selected to represent the postfusion form of the HCMV gB ectodomain in subsequent studies. When produced in 293-6E cells with a C-terminal HisAvi tag (S1A and S1B Fig), pEW2 adopted the postfusion form, judging by NS-EM (Figs 2B, 2C and S1C).

Next, to destabilize the postfusion form, we introduced helix-disrupting mutations into the long central helix in domain III (Fig 1C). Previously, single helix-disrupting Pro mutations failed to destabilize the postfusion HSV-1 gB ectodomain [60,61]. Likewise, multiple Pro mutations yielded only the postfusion form (S1 Table). To destabilize the central helix more drastically, we replaced large sections with residues that favor intrinsically disordered regions (Pro, Glu, Ser, Lys, and Gln) [reviewed in [62,63]] (S1 Table). One construct in this series, “pEW21”, in which residues 480–493 were replaced with Gly, Thr, or Ser (GTS mutations) (Fig 2D and S1 Table), was produced in 293-6E cells with a C-terminal HisAvi tag (S1A and S1B Fig) and examined by NS-EM. While 30% of pEW21 adopted a postfusion conformation, 70% adopted various intermediate conformations (Figs 2E, 2F, S1D and S1E). This construct was used in subsequent studies to represent the intermediate form of the HCMV gB ectodomain.

We later attempted to improve our GS mutations by replacing a larger section of the central helix (residues 478–501) with residues that favor intrinsically disordered regions. This mutant, termed “eDDR2” (Fig 2G), yielded a mixture of prefusion, intermediate, and postfusion forms by NS-EM (S1 Table). To further stabilize the prefusion form, we introduced interprotomer cysteine (Cys) pairs predicted to form disulfides in the prefusion structures of HSV-1 gB [61] (S1 Table). Pairs of residues were selected using a standard design approach to promote disulfide formation, i.e., ensuring favorable Cb-Cb distance and the chiral angle [64]. The HCMV postfusion gB structure was also analyzed to ensure that the designed disulfides could not form in the postfusion form. To avoid potential misfolding, new Cys were placed away from existing disulfides whenever possible. Several resulting constructs were found to preferentially adopt the prefusion conformation as assessed by NS-EM (S1 Table). One of these, pEW62 (Fig 2G), was selected for downstream characterization and was produced in 293-6E cells with a C-terminal HisAvi tag (S1A and S1B Fig) and examined by NS-EM. Most of pEW62 (~85% of particles) adopted a conformation that matched the known structure of the native HCMV prefusion [24], with a small minority (~15% of particles) in the postfusion form [25,26] (Figs 2H, 2I and S1F). pEW62 was used in subsequent studies to represent a variant of the prefusion form of the HCMV gB ectodomain.

In addition to NS-EM analysis, we also directly compared the behavior of pEW2, pEW21, and pEW62 across a series of biochemical quality control experiments (S1 Fig). All three constructs showed nearly identical mobility in size-exclusion chromatography (S1A Fig). Each construct migrated as two prominent bands under reducing SDS-PAGE conditions (S1B Fig), consistent with efficient cleavage of HCMV gB by host furin [65,66]. The lower molecular weight band corresponding to the C-terminal fragment of gB showed comparable mobility for pEW21 and pEW2, while the C-terminal fragment of pEW62 was retarded, likely due to the extensive amino acid differences introduced to stabilize the pre-fusion state (S1B Fig). Under non-reducing conditions, pEW21 and pEW2 migrated as individual protomers (~130 kDa) whereas pEW62 migrated more slowly confirming efficient inter-protomer disulfide linking (S1B Fig). Each construct bound fragment antigen-binding regions (Fabs) generated from known anti-HCMV gB human mAbs with defined epitopes within different domains of gB – 1G2 (dI, AD-5) [30] and SM5–1 (dII, AD-4) [30] – with high affinity (S1G and S2 Figs and S2 Table).

### None of the conformational forms of the HCMV gB ectodomain elicit neutralizing antibodies

Like CMV gB, the RSV fusion protein, F can assume structurally divergent pre- and post-fusion states, the latter of which is the major target of neutralizing antibodies [46] and forms the basis for recently approved RSV vaccines [39,45,50]. Therefore, we sought to compare the immunogenicity of the prefusion-stabilized HCMV gB variant (pEW62) with the postfusion HCMV gB (pEW2). To our knowledge, the immunogenicity of intermediate gB has not been previously tested; therefore, we also included our intermediate HCMV gB construct (pEW21) in these immunization studies.

**Fig 3:**
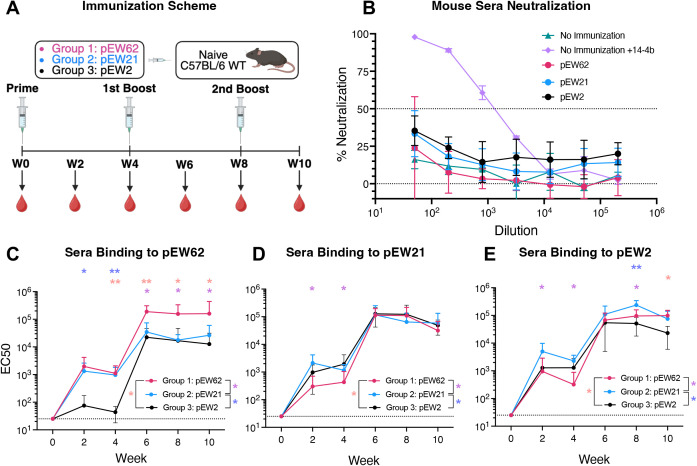
Immunization with gB variants does not elicit neutralizing antibodies in mice. **(A)** HCMV gB immunization scheme and timeline. Bleeds were collected retro-orbitally at the indicated time points. **(B)** Neutralization of sera from all immunized mice against AD169_GFP_. Sera from mice, n = 7 (pEW62) or n = 5 (pEW21, pEW2), were tested individually in technical duplicate. Sera from naïve mice were used as a negative control. Sera from naïve mice with known nAb 14-4b were used as a positive control. 14-4b was added to the sera at a starting concentration of 100 μg/mL and serially diluted with the sera as indicated. Symbols represent the mean, and error bars represent the standard deviation of each group, n = 7 (pEW62), n = 5 (pEW21, pEW2), n = 3 (negative control, positive control) in B-D. **(C-E)** Half-maximal effective concentrations (EC_50_) of serum antibody binding endpoint titers to **(C)** pEW62, **(D)** pEW21, and **(E)** pEW2 as measured by ELISA. Samples were grouped by immunization group and are colored in accordance with the color scheme defined in **(A)**. Sera from naïve mice were used as controls. ** indicates P < 0.01, and *, P < 0.05, as determined by Mann-Whitney tests. Stars are colored in accordance with the groups being compared, as shown in (D). *Fig 3a. Immunization Scheme. Created in BioRender. McClave, M. (2025)* *https://BioRender.com/yipgib3*.

pEW62, pEW21, and pEW2, produced in 293-6E cells with C-terminal HisAvi tags, were formulated with adjuvant, and used to immunize groups of 7 (pEW62 immunization) or 5 (pEW21 and pEW2 immunizations) C57BL/6 mice three times at weeks 0, 4, and 8 (Fig 3A). Diluted terminal sera were tested for neutralization against HCMV AD169_GFP_. Serum from naïve mice spiked with the known anti-HCMV nAb 14-4b [67] was included as a control. Undiluted sera and dilutions lower than 1:50 resulted in cytotoxicity and an artificial increase in GFP signal and were, therefore, not tested. None of the sera from the immunized mice achieved 50% neutralization, which we consider a minimum threshold for true neutralizing activity (Fig 3B), unlike the serum spiked with the 14-4b control, which achieved 100% neutralization and showed a dose-dependent effect (Fig 3B).

We measured the serum antibody binding to all three gB constructs by ELISA. Mice immunized with pEW62 showed the strongest binding to pEW62, followed by mice immunized with pEW21 and mice immunized with pEW2 (Fig 3C). The lower titers elicited by pEW2 were more pronounced after the first immunization and less noticeable after the second immunization. Mice immunized with pEW21 reacted strongest to pEW21 until week 8, then dropped to the level of the pEW62 immunized group (Fig 3D). Mice immunized with any of the three gB variants showed comparable binding to pEW2 after two immunizations (Fig 3E). Thus, all three constructs of gB were immunogenic and elicited Abs that bound to all three gB conformations, with distinct binding preferences.

In summary, all three constructs were immunogenic, eliciting high-titers of binding antibodies, and were antigenically distinct. However, none elicited measurable nAbs, demonstrating that stabilization of HCMV gB in prefusion conformation does not improve its ability to elicit nAbs. While contrary to studies performed with RSV F, these findings are in-line with other recent studies that showed that immunization with prefusion HCMV gB did not outperform postfusion HCMV gB [53,68]. Similar observations have been reported for HSV-1 gB [69].

### mAb isolation and confirmation of binding to HCMV gB

**Fig 4:**
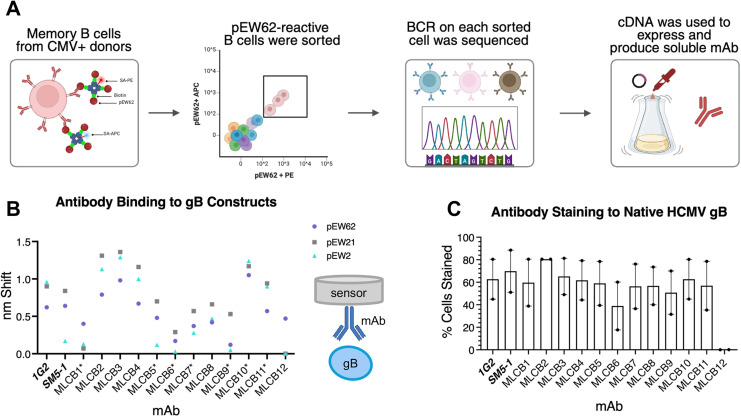
mAb isolation and binding assessment to all conformations of gB. **(A)** mAb isolation strategy. Enriched human B cells were stained with fluorescently labeled pEW62. The antibody variable heavy and light chain transcripts were recovered by RT-PCR, cloned into expression vectors, and produced as recombinant mAbs. For full gating strategy, see S3 Fig. **(B)** Binding avidity of the indicated mAbs to pEW62, pEW21, or pEW2 at a single concentration was measured by biolayer interferometry using isolated antibodies immobilized on biosensors and indicated gB construct as the ligand. Each data point represents the maximum binding response (nm shift) response of each mAb to a 40 µg/mL solution of each of the three constructs after 150 seconds. Data are representative of one independent experiment. **(C)** Percent gB detected on cells. Cells were transfected with AD169 gB, permeabilized, and tested for % gB staining by the indicated mAb. Each point represents an independent transfection. Bar heights represent the mean, and the error bars represent the standard error of the mean (SEM). For full gating strategy, see S4 Fig. *Fig 4a. mAb isolation strategy. Created in BioRender. McClave, M. (2025)* *https://BioRender.com/bzak0d0*.

In light of immunogenic differences between pEW62 (Fig 3C) relative to pEW21 and pEW2 (Fig 3D and 3E), we asked whether the pEW62 construct could be used to isolate conformation-specific antibodies arising from natural infection. To this end, we conjugated pEW62 to two different fluorophores and labeled IgG + B cells from CMV seropositive donors (Figs 4A and S3). A decoy protein conjugated to yet another fluorophore was also included. This strategy allowed us to more reliably identify rare, antigen-specific B cells [70,71]. We sorted 52 decoy-negative, pEW62-dual-positive B cells, from which we recovered 12 paired heavy and light chain transcripts, which we named MLCB1-MLCB12. All represented unique rearrangements (S3 Table). When produced as recombinant monoclonal antibodies (mAbs), all bound to the pEW62 probe used for sorting, and only MLCB9 had weak reactivity (Fig 4B). Most mAbs could also bind other forms of gB represented by pEW21 and pEW2 (Fig 4B) and, used at a single concentration, displayed varying degrees of reactivity to each construct. For example, MLCB9 had weak reactivity to pEW62. MLCB1 and MLCB12 had weak reactivity to pEW21 (Fig 4B). MLCB1, MLCB5, MLCB6, MLCB9, and MLCB12 had weak reactivity to pEW2 (Fig 4B).

To test for the ability of the MLCB mAbs to bind native HCMV gB, we overexpressed wild-type HCMV AD169 gB in 293-6E cells, permeabilized them, and stained with each antibody. Known anti-HCMV gB human mAbs 1G2 [30] and SM5–1 [30] were used as controls. All antibodies except MLCB12 bound permeabilized transfected cells (Figs 4C and S4).

### Seven out of 12 new mAbs neutralize at least one HCMV strain

**Fig 5:**
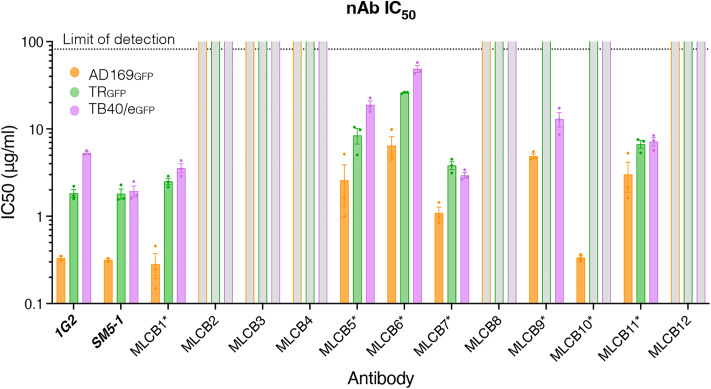
Seven antibodies neutralize at least one HCMV strain. HCMV with GFP transgene (AD169_GFP_ MOI = 30, TR_GFP_ & TB40/e_GFP_ MOI = 5) was incubated alone or with the antibody of interest for two hours before being added to confluent HFFs. Cells were then incubated with the mAb/virus mixture for 18 hours (AD169_GFP_) or 48 hours (TR_GFP_ and TB40/e_GFP_), then washed and fixed with 4% PFA. The plate was read in a microplate reader for bulk GFP intensity. Half-maximal inhibitory concentration (IC_50_) values for antibodies identified as neutralizing for one or more strains (AD169_GFP_ orange, TR_GFP_ green, TB40/e_GFP_ purple). nAbs were titrated against each indicated strain of HCMV, and IC_50_ was calculated from the resulting linear regression (see S6 Fig). Each data point is an average of three technical replicates. Each experiment was done in triplicate. Gray bars were used to denote that an IC_50_ for an indicated strain/mAb was above the limit of detection of our assay (82 μg/mL).

To characterize the neutralization properties of the MLCB mAbs, we established a fluorescence-based neutralization assay for the HCMV AD169_GFP_ [72], TR_GFP_ [73], and TB40/e_GFP_ [73] strains (Figs 5 and S5). All 12 mAbs were first tested at a single high concentration, 100 mg/mL (S5 Fig). This concentration is 300 times higher than the published IC_50_ values of the control Abs, 1G2 (0.2 mg/mL) and SM5–1 (0.3 mg/mL) [30], and was used to detect even weak neutralizers. Abs that showed greater than 60% neutralization at this concentration were titrated (S6 Fig) as this would place their putative IC_50_ at the limit of detection by our assay (82 mg/mL).

Of the 12 mAbs, seven (MLCB1, MLCB5, MLCB6, MLCB7, MLCB9, MLCB10, and MLCB11) neutralized AD169_GFP_ (Figs 5 and S5). As AD169_GFP_ is lab-adapted, we also tested these mAbs against two minimally passaged strains of HCMV, TR_GFP_ and TB40/e_GFP_ [73]. Of the seven mAbs that neutralized AD169_GFP_, six (MLCB1, MLCB5, MLCB6, MLCB7, MLCB9, and MLCB11) neutralized TB40/e_GFP_ (Figs 5 and S5) whereas five (MLCB1, MLCB5, MLCB6, MLCB7, and MLCB11) also neutralized TR_GFP_ (Figs 5 and S5).

The seven neutralizing mAbs had IC_50_ values that ranged from 0.284 mg/mL to 6.46 mg/mL (Fig 5 and Table 1). Generally, the new mAbs neutralized AD169_GFP_ more potently than TR_GFP_ or TB40/e_GFP_. The most potent neutralizer, on par with control mAbs 1G2 and SM5–1, that neutralized all three HCMV strains was MLCB1 (Figs 5 and S5 and Table 1). MLCB10 also had strong neutralizing potency but only against AD169 (Figs 5 and S5 and Table 1). Thus, we have successfully isolated several potent, broadly neutralizing mAbs

**Table 1:** IC_50_ values for the nAbs tested in this study.

nAb	IC_50_, AD169_GFP_ (μg/mL)	IC_50_, TR_GFP_ (μg/mL)	IC_50_, TB40/e_GFP_ (μg/mL)
*1G2*	0.33 + /-0.02	1.83 + /-0.19	5.30 + /-0.18
*SM5–1*	0.32 + /-0.01	1.81 + /-0.24	1.95 + /-0.28
MLCB1	0.28 + /-0.09	2.51 + /-0.21	3.57 + /-0.430
MLCB5	2.56 + /-1.30	8.43 + /-1.71	18.91 + /-2.04
MLCB6	6.46 + /-1.71	26.0 + /-0.36	48.82 + /-4.46
MLCB7	1.09 + /-0.18	3.82 + /-0.40	2.96 + /-0.23
MLCB9	4.89 + /-0.31	NN	12.93 + /-2.50
MLCB10	0.34 + /-0.02	NN	NN
MLCB11	3.02 + /-1.14	6.70 + /-0.69	7.16 + /-0.79

IC_50_ and SEM were quantified for each nAb against the indicated strain from the titration curves in S6 Fig. These values were also plotted in Fig 5. NN (non-neutralizing) was used to denote that an IC_50_ for an indicated strain/mAb was above the limit of detection of our assay (82 μg/mL).

### The antibodies vary in their conformational specificity

**Fig 6:**
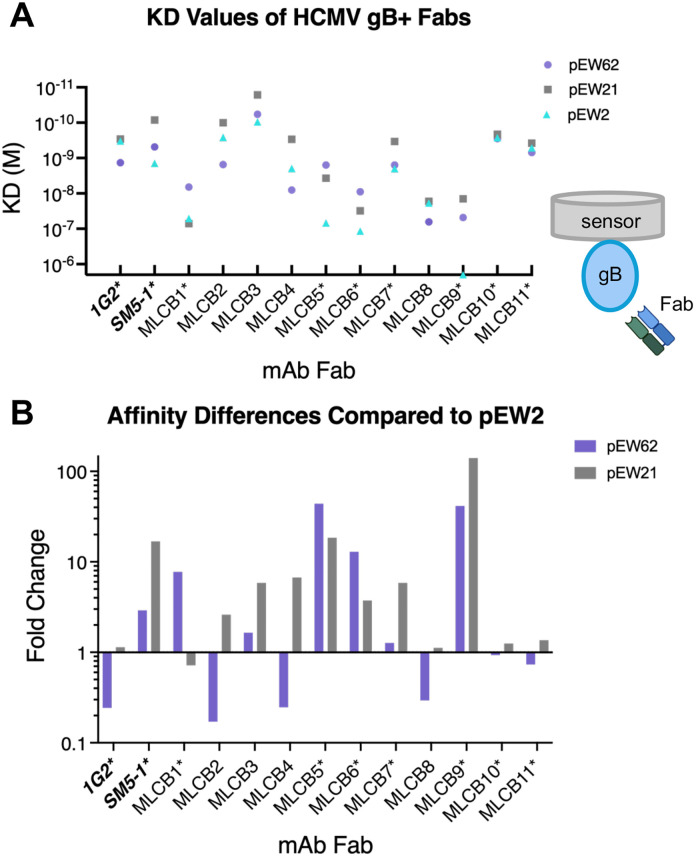
Antibodies show diverse preferences for different gB constructs. **(A)** Monovalent affinities of the indicated Fabs to pEW62, pEW21, and pEW2 were measured by BLI. KDs were calculated from multiple measurements of serially diluted Fab. For full kinetic curves, see S2 Fig. Data for 1G2 and SM5-1 are the same as in S1G Fig. MLCB12 did not bind any of the constructs in Fab form and thus was excluded from analysis. **(B)** Fold-change differences in Fab affinity across gB constructs. To visualize relative shifts in binding strength, for each Fab, the KD values in (A) were transformed into fold-change differences by normalizing the KD values for pEW62 and pEW21 to that of pEW2. Relative affinity for pEW62 is shown in purple, relative affinity for pEW21 in gray, corresponding to coloring of constructs in panel **(A)**. Fold-change was calculated as (KD_pEW2_)/(KD_pEW62_ or KD_pEW21_). Values >1 indicate increased affinity relative to pEW2, values <1 indicate reduced affinity relative to pEW2. For KD values, see S2 Table.

To assess the conformational specificity of mAbs, we measured the monovalent binding affinity of their Fabs (Figs 6A and S2 and S2 Table). To visualize binding differences, for each Fab, we normalized the KD values for pEW62 and pEW21 to that of pEW2 and expressed these as fold-changes (Fig 6B). The postfusion construct, pEW2, was chosen as the normalization standard, as previous HCMV gB antibody studies were limited to the postfusion conformation. This re-expression of data allowed us to highlight preferential recognition patterns and relative sensitivities of each Fab to different gB conformations. Given that pEW21 is a mixture of intermediate and postfusion gB species, any minor differences in mAb specificity to pEW21 or pEW2 should be interpreted as higher in magnitude than reported. Likewise, the minority postfusion population in pEW62 would alter the magnitude of difference between constructs, favoring pEW62 over pEW2. Several clear visual patterns became evident from our data.

Of all tested Fabs, only MLCB1 showed a binding preference for pEW62 over the other two constructs (Figs 6 and S2 and S2 Table). MLCB5 and MLCB6 bound pEW62 slightly better than pEW21 and better than pEW2. SM5–1, MLCB3, MLCB4, MLCB7, and MLCB9 had a binding preference for pEW21 over pEW62 and pEW2 (MLCB9 did not bind pEW2). 1G2 and MLCB8 bound pEW21 and pEW2 comparably and better than pEW62. MLCB10 and MLCB11 had comparable affinities to all three constructs. MLCB12 Fab did not bind to any of the constructs. The conformational preferences varied across the mAbs (Figs 6 and S2 and S2 Table) and did not appear to correlate with neutralization efficiency (Figs 5 and S5). Thus, despite being isolated by virtue of binding pEW62, most mAbs bind epitopes present on all forms of gB.

### Competition analysis identifies six distinct epitope clusters

**Fig 7:**
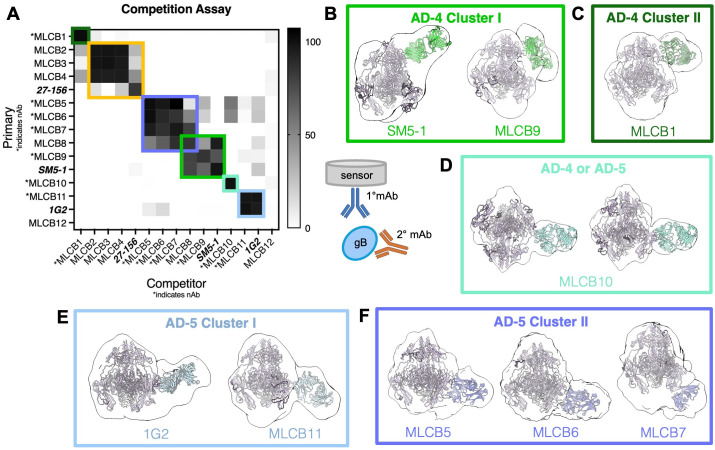
Neutralizing antibodies bind to HCMV gB AD-4 and AD-5 and form five distinct clusters. **(A)** BLI competition assay. Biotinylated mAbs were immobilized on streptavidin biosensors (y-axis) and then immersed in buffer containing pEW62 with or without the indicated non-biotinylated mAb (x-axis). The percent binding inhibition is shown as a heatmap. **(B-F)** Models of the prefusion gB ectodomain bound to the corresponding Fab were generated and manually fitted into the NS-EM reconstructions of pEW62/Fab complexes as described in the Materials and Methods. For representative particle images, see S9 Fig. Complexes were subdivided into clusters based on competition assay data in (A). The pEW62/MLCB10-Fab complex in (D) could not be conclusively assigned to AD-4 or AD-5, due to map symmetry and lack of competitors. For detailed information on map and model fitting, see Materials and Methods.

To locate the epitopes recognized by the mAbs, we first assessed their ability to compete for binding to pEW62 (Fig 7A). Three known anti-HCMV gB mAbs with defined epitopes within different domains of gB were chosen as reference: human 1G2 (dI, AD-5) [30], human SM5–1 (dII, AD-4) [30], and mouse 27–156 (dIV, AD-1) [74]. Competition analysis revealed that eleven new mAbs and three reference mAbs formed six distinct epitope sites (Fig 7A). MLCB12 did not compete with itself or any other mAb.

MLCB1 and MLCB10 did not compete with any other mAbs and, therefore, likely target unique epitopes (Fig 7A). Non-neutralizing mAbs MLCB2, MLCB3, and MLCB4 competed with each other and, to a lesser extent, with 27–156. Therefore, their epitopes are likely located within AD-1 (dIV). MLCB5, MLCB6, and MLCB7 competed with each other and, to a lesser extent, with MLCB8. MLCB8 also competed with MLCB9 and with SM5–1, which suggests that MLCB8 and MLCB9 bind epitopes within AD-4 (dII). MLCB11 competed with 1G2 and likely binds an epitope within AD-5 (dI).

### mAbs bind linear and conformational epitopes

To determine whether the mAbs bound to linear or conformational epitopes, we probed their ability to bind denatured and reduced pEW62 by SDS-PAGE and Western Blot (S7 Fig). HCMV gB is post-translationally cleaved by furin between dII and dIII, which generates a larger N-terminal (dI and dII) and a smaller C-terminal fragment (dIII, dIV, and dV), covalently linked by five disulfides (Fig 1A). On a reducing SDS-PAGE, pEW62 migrated as two bands at ~110 kDa (N-terminal fragment) and ~55 kDa (C-terminal fragment) (S7A Fig). MLCB1, MLCB7, and SM5–1 bound linear epitopes within the ~ 110-kDa N-terminal fragment. Despite competing for binding with SM5–1 (Fig 7A), MLCB8 and MLCB9 were not reactive by Western Blot (S7B Fig), suggesting that they bind conformational epitopes that overlap that of SM5–1. Similarly, MLCB5 and MLCB6 were not reactive by Western Blot (S7B Fig) and likely bind conformational epitopes that overlap that of MLCB7. MLCB2 and MLCB3 bound a linear epitope within the ~ 55-kDa C-terminal fragment, whereas their competitors, MLCB4 and 27–156, did not (S7B Fig). The remaining mAbs showed no reactivity to denatured pEW62, indicating they bind conformational epitopes on gB (S7B Fig).

### nAbs bind distinct sites in antigenic domains 4 and 5 of gB

To visualize the binding locations of the nAbs, we employed Negative-Stain Electron Microscopy (NS-EM). Known antibodies 1G2 and SM5–1 were included to validate the analysis pipeline. To prepare complexes for NS-EM, pEW62 was incubated with a 2-molar excess of Fab. The pEW62/Fab complexes were separated from unbound Fab by size-exclusion chromatography (S8 Fig), cross-linked with glutaraldehyde, and applied to grids. In all cases, including control antibodies, we observed sub-stoichiometric binding of Fabs to pEW62 (S9 Fig). Most particles corresponded to pEW62 bound to a single Fab, some had two Fabs, or, in rare instances, three Fabs (S9 Fig).

The 3D NS-EM reconstructions captured this sub-stoichiometric binding, with a single Fab bound to pEW62. All nAbs appeared to bind epitopes in either AD-4 (dII) or AD-5 (dI) (Fig 7B–7F). MLCB1 and MLCB9 appeared to bind AD-4, just as SM5–1 (Fig 7B–7D). Based on the competition data (Fig 7A), however, they were assigned to two distinct clusters within AD-4: AD-4 Cluster I (MLCB9 and SM5–1) (Fig 7B) or AD-4 Cluster II (MLCB1) (Fig 7C).

MLCB10 presented an interesting case because the symmetrical nature of its NS-EM and lack of competitors resulted in its epitope tentatively assigned to either AD-4 or AD-5 (Fig 7D). High-resolution structure information will be required to locate its epitope more accurately.

MLCB5, MLCB6, MLCB7, MLCB11 and 1G2 all bound AD-5 (dI) and were separated into two clusters: AD-5 Cluster I (MLCB11 and 1G2) (Fig 7E) and AD-5 Cluster II (MLCB5, MLCB6, and MLCB7) (Fig 7F). The nomenclature, “cluster”, was used to serve as a precursor for identification of Sites within AD-4 and AD-5, in line with previously established Sites I and II of AD-2 [33]. These aforementioned Sites correspond to specific residues within AD-2, and, as our low-resolution data cannot map the specific epitopes, we aim for the clusters to serve as a guiding basis for downstream high-resolution mapping of AD-4 and AD-5 Sites. The binding domain locations deduced from the NS-EM reconstructions were consistent with the competition data (Fig 7A).

MLCB9 and SM5–1 compete for binding to AD-4 Cluster I (Fig 7A and 7B). But whereas SM5–1 binds a discontinuous linear epitope including residues 359–362 and 379–383 [75], MLCB9 binds a conformational epitope (S7 Fig). The MLCB9 epitope could, in principle, include residues 359–362 or 379–383. However, unlike SM5–1, which neutralized all three HCMV strains, MLCB9 neutralized AD169 and TB40/e strains but not TR (Figs 5 and S5 and Table 1). The only residue in AD-4 (dII) conserved in AD169 and TB40/e but not in TR is 462 (T462 in AD169 and TB40/e and A462 in TR). Therefore, T462 likely lies within the epitope of MLCB9 (S10C Fig). The SM5–1 epitope does not include residue 462 (S10A and S10C Fig). Thus, MLCB9 and SM5–1 could compete sterically.

MLCB10 neutralized only AD169 but not TR or TB40/e (Figs 5 and S5 and Table 1) and binds a conformational epitope in AD-4 or AD-5 (Figs S7 and 7D). Both AD-4 and AD-5 have various regions of sequence diversity between the three strains, so unlike MLCB9, we are unable to pinpoint its epitope from sequence analysis (S10 Fig).

### Some nAbs cannot access their epitopes on cell surface

**Fig 8:**
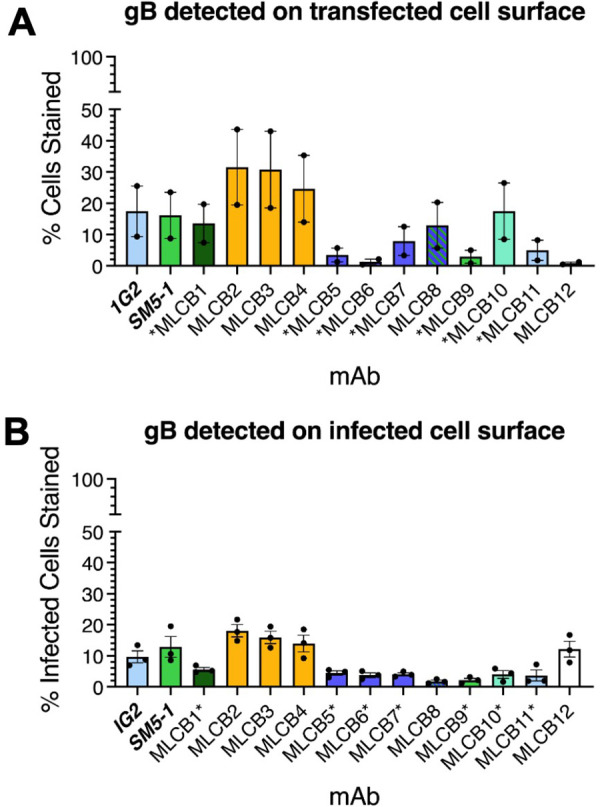
Not all neutralizing antibodies can access their epitopes on the cell surface. **(A)** Percent gB detected on the surface. Cells were transfected with AD169 gB and tested for percent gB staining by the indicated mAb. Each point represents an independent transfection. Bar heights represent the mean, and the error bars represent SEM. See S11 Fig for data on individual replicates. MLCB12 could not be calculated due to a lack of binding. **(B)** Percent gB detected on the surface of cells infected with AD169_GFP_. Cells were infected with AD169_GFP_ at an MOI of 3 and stained with the indicated mAb. Results indicate infected cells (GFP+) that were positive for each mAb. Each point represents an independent infection. Bar heights represent the mean, and the error bars represent SEM. For full gating strategy, see S12 Fig. Antibodies are colored according to their binding sites in Fig 7.

To determine whether the mAbs could access their epitopes on cell surface, we assessed their staining of non-permeabilized 293-6E cells transiently transfected with HCMV AD169 gB construct by flow cytometry (Figs 8A and S11). mAbs with poorly accessible epitopes would be expected to have lower levels of cell-surface staining. Conversely, mAbs with readily accessible epitopes would be expected to have higher level of cell-surface staining. Overall, fewer cells stained positive when they were not permeabilized (Figs 8A and S11) relative to when they were (Figs 4C and S4), consistent with the presence of an endocytic recycling motif on the cytoplasmic domain of gB [76].

We observed high surface staining with the non-neutralizing mAbs MLCB2, MCLB3, and MLCB4 (Fig 8A), ~ 30%, which have putative epitopes in AD-1 (dIV). Except for 1G2, cell-surface staining with mAbs targeting AD-5 (dI) was low, 5–10% (Fig 8A), which is consistent with the membrane-proximal location of dI in both prefusion and postfusion forms of gB (Fig 1B and 1C). 1G2 had higher staining, ~ 15% (Fig 8A), possibly because its epitope is located within the upper subdomain of dI that is more readily accessible. Except for MLCB9, mAbs targeting AD-4 (dII) had a relatively high percentage of gB surface staining, ~ 15–20% (Fig 8A), consistent with the distal location of dII relative to the membrane in both prefusion and postfusion forms of gB (Fig 1B and 1C). The reason for low cell-surface staining with MLCB9 is yet unclear.

To assess mAb binding to the surface of infected cells, we analyzed mAb staining of HFF cells infected with AD169_GFP_ by flow cytometry (Figs 8B and S12). Although the level of cell-surface staining of infected cells was lower overall (Fig 8B), relative staining showed similar tendencies to transfected cells (Fig 8A). Again, non-neutralizing, AD-1 (dIV) mAbs MLCB2, MCLB3, and MLCB4 showed the highest staining, ~ 15–20%. Except for 1G2, cell-surface staining with mAbs targeting AD-5 (dI) was very low (Fig 8B). Just as in transfected cells, cell-surface staining with AD-4 (dII)-targeting mAb SM5–1 was relatively high and low with MLCB9 (Fig 8B). However, staining with other AD-4 (dII)-targeting mAbs, MLCB1 and MLCB10 was noticeably lower in infected (Fig 8B) vs. transfected cells (Fig 8A). We hypothesize that their epitopes may overlap the binding site for gH/gL. Alternatively, gH/gL binding to gB could prevent mAb binding through steric hindrance or allostery. This hypothesis could also account for low staining in infected cells observed with non-neutralizing mAb MLCB8 (Fig 8B), the epitope of which likely spans AD-5 (dI) and AD-4 (dII) (Fig 7A). MLCB12 showed relatively high staining in infected cells (Fig 8B) relative to transfected cells (Fig 8A). However, given that this mAb did not compete with itself (Fig 7A) and did not stain either permeabilized (Fig 4C) or non-permeabilized cells transfected with HCMV gB (Fig 8A), the observed staining could be non-specific.

HCMV gB has been found in both prefusion and postfusion conformations on the virion surface [77]. Therefore, both forms may also exist on the surface of infected or transfected cells. Indeed, HSV-1 gB adopts both forms on the surface of transfected cells [61,78,79]. The ratios of prefusion to postfusion form on the cell surface is yet unknown and may be different for infected vs. transfected cells. Regardless, AD-5 (dI)-targeting nAbs MLCB5, MLCB6, MLCB7, and MLCB11 poorly stain the surface of either infected or transfected cells (Fig 8B), because in both pre- and postfusion forms, their epitopes are proximal to the membrane and inaccessible. Therefore, we hypothesize that these antibodies could neutralize HCMV by binding gB intermediates.

## Discussion

### Proposed neutralization mechanisms of nAbs investigated here

**Fig 9:**
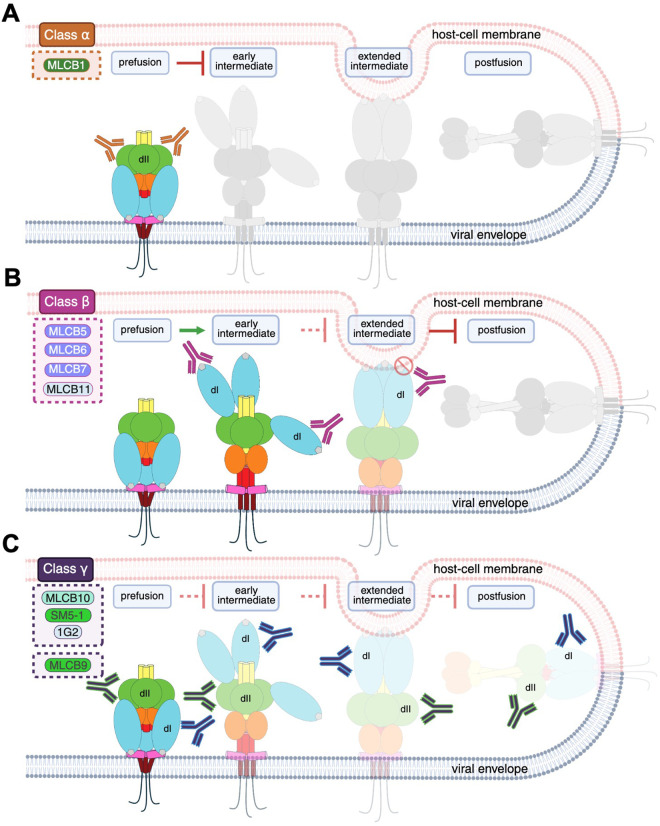
Putative mechanisms of nAbs described in this study. Schematic illustrations representing prefusion, early intermediate, extended intermediate, and postfusion HCMV gB are shown to illustrate possible binding locations of nAbs. Domains of gB are colored in accordance with the color scheme defined in Fig 1. Fusion loops are represented by gray pentagons on the tips of dI. Domains bound by nAbs of a particular class are additionally labeled for clarity. Members of each class were determined by combining data from competition assay, cell surface staining, NS-EM reconstructions, and affinity measurements. **(A)** Class α consists of MLCB1, shown in orange square on the left. **(B)** Class β consists of the majority of the nAbs isolated here, MLCB5, MLCB6, MLCB7, and MLCB11, shown in berry-colored box on the left. Red prohibition circle on extended intermediate form represents the inhibition of membrane binding or the refolding of the intermediate into the postfusion form. **(C)** Class γ consists of MLCB10, SM5-1, 1G2, along with MLCB9. These are shown in a purple box on the left. Blue and green antibody borders represent dI and dII binders, respectively. *Created in BioRender. McClave, M. (2025)* *https://BioRender.com/xydn9a1*.

Prefusion-specific antibodies targeting viruses such as RSV are potent neutralizers *in vitro* and protective *in vivo* [39,45]. Here, we engineered a soluble, prefusion-stabilized HCMV gB variant (pEW62) and used it to isolate gB-specific B cells that yielded twelve new human mAbs. Seven of these mAbs neutralized at least one HCMV strain whereas six neutralized at least two different HCMV strains, i.e., were broadly neutralizing. MLCB1, which preferentially bound the prefusion over postfusion and intermediate forms, emerged as one of the most potent neutralizers. This finding supports our hypothesis that antibodies preferentially binding prefusion form may exhibit robust neutralization, potentially by stabilizing the prefusion conformation and preventing its refolding. However, many mAbs – including nAbs – either preferentially bound the intermediate or had no strong conformational preference. These data suggest that neutralization can be achieved by mechanisms that target other gB conformations, i.e., the intermediate. Indeed, by analyzing conformational preference, reactivity with gB on the cell surface, and epitope location, we separated the seven nAbs isolated here, along with 1G2 and SM5–1, into three mechanistic classes based on their putative neutralization mechanisms (Fig 9).

Class α consists of MLCB1 (Fig 9A). MLCB1 preferentially binds prefusion form (Fig 6), does not compete with other mAbs (Fig 7A), and binds a linear epitope (S7B Fig) within AD-4 (dII) (Fig 7C) that is accessible on the surface of gB-transfected cells (Fig 8A) and less accessible on the surface of HCMV-infected cells (Fig 8B). We hypothesize that MLCB1 neutralizes HCMV by binding to the prefusion form of gB and blocking its fusogenic refolding (Fig 9A).

Class β consists of the AD-5 (dI)-targeting nAbs isolated here, MLCB5, MLCB6, MLCB7, and MLCB11 (Fig 9B). Their epitopes on AD-5 (dI) (Fig 7E and 7F) are poorly accessible on the cell surface of both gB-transfected and HCMV-infected cells (Fig 8A and 8B). We hypothesize that these nAbs cannot access their epitopes on the virion surface in either prefusion or postfusion form and, instead, bind the refolding intermediates of gB, in which AD-5 (dI) would be accessible (Fig 9B). Class β nAbs may neutralize the virus by binding the intermediate forms and either by blocking the refolding of an intermediate conformation into the postfusion form or by blocking fusion loops from interacting with the target membrane through steric hindrance (Fig 9B). We hypothesize that Class β nAbs could even promote prefusion-to-intermediate refolding by preferentially binding early intermediates.

Class γ consists of MLCB10, SM5–1, and 1G2 (Fig 9C). These three antibodies bind to all three gB forms, prefusion, intermediate, and postfusion (Fig 6). Their epitopes in AD-4 (dII) for MLCB10 and SM5–1 or AD-5 (dI) for MLCB10 and 1G2 (Fig 7B, 7D and 7E) – are accessible on the surface of gB-transfected cells (Fig 8A) and, with the exception of MLCB10, accessible on the surface of HCMV-infected cells (Fig 8B). Thus, we hypothesize that class γ antibodies neutralize HCMV by binding prefusion and intermediate forms and blocking gB refolding at different steps (Fig 9C). This hypothesis is in agreement with a previously proposed mechanism of action for SM5–1, wherein the nAb prevents the transition to postfusion form [75].

MLCB9 was also assigned to class γ, yet it presents a special case (Fig 9C). MLCB9 does not exhibit a preference for prefusion over the intermediate but does not bind the postfusion form (Fig 6). Therefore, we hypothesize that MLCB9 binds prefusion and intermediate forms and blocks their refolding to the postfusion form (Fig 9C).

### mAbs isolated in this study have potential downstream applications

Here, we have isolated twelve new human mAbs from HCMV-seropositive donors. Seven of these mAbs strongly neutralized at least one strain of HCMV *in vitro* whereas six mAbs neutralized both lab-adapted and minimally passaged clinical strains. All nAbs bound different epitopes within antigenic regions AD-4 or AD-5, and most targeted new sites. The nAbs had distinct conformational specificity and different neutralization mechanisms. Despite being isolated using a prefusion-stabilized HCMV gB variant (pEW62), nAbs varied in their conformational specificity and presumed neutralization mechanisms. Only one nAb preferentially bound the prefusion form and most bound the intermediate form. Thus, HCMV gB has multiple neutralization-sensitive epitopes that are targeted by potent nAbs that arose during natural infections. We hypothesize that these nAbs and bnAbs can be developed into prophylactics to prevent congenital CMV infections in neonates or therapeutics to prevent CMV morbidities in the immunocompromised individuals such as solid-organ transplant recipients. Antibody engineering approaches could improve potency and breadth of neutralization, half-life, and effector functions of the mAbs. The use of such mAbs, with defined activity and pharmacokinetics, may prove more efficacious than current polyclonal IgG treatments.

While we have identified the domain targets of each new nAb, we have not yet mapped their precise epitopes. Nonetheless, we have been able to narrow down the epitope of MLCB9 nAb that did not neutralize all three strains of HCMV we tested – with the help of sequence alignments. Future structural studies of gB/Fab complexes will map their epitopes more precisely, aiding in developing gB-based immunogens.

Three of the non-neutralizing mAbs, MLCB2, MLCB3, and MLCB4, compete with known murine non-neutralizing mAb 27–156 (Fig 7A) [80] that binds AD-1 (dIV) [81]. Downstream structural comparison of MLCB2, MLCB3, MLCB4, and 27–156 may clarify why these antibodies do not neutralize despite being able to bind different forms of gB in solution and show high surface staining levels in both gB-transfected and HCMV-infected cells (Fig 8A and 8B). Additionally, a recent study suggested that non-neutralizing AD-1 (dIV)-targeting mAbs could be important for ADCP [82]. Therefore, MLCB2, MLCB3, MLCB4, and the remaining mAbs should be further investigated for their ability to mediate effector functions.

### pEW62 has potential for use in vaccine development and antibody discovery

The prefusion-stabilized HCMV gB variant ectodomain developed here (pEW62) has potential for both vaccine development and therapeutic antibody discovery. By engineering a soluble, stable prefusion variant of HCMV gB, we have achieved a significant improvement over some previous stabilization methods, which were limited by low prefusion stability and the use of small molecules and detergents that may mask potential epitopes [24].

Isolation of twelve new mAbs, seven of which were neutralizing and six were broadly neutralizing, validates pEW62 for downstream use in additional mAb isolation studies and immunogen design. Nonetheless, our NS-EM reconstructions also suggest how prefusion-stabilized constructs based on pEW62 could be improved. NS-EM data for each of the pEW62/nAb complexes described in this study displayed sub-stoichiometric binding of Fab to gB. We hypothesize that this is due to an increased flexibility in dI and dII of pEW62 as a consequence of our stabilization methods. Our eDDR2 mutations likely successfully destabilized the central helix of gB dIII, but in doing so, may have allowed more lateral movement of dII. The location of our disulfides could have perturbed the native location of dI relative to dIV, increasing the vertical movement of dI. Studies are underway to examine these hypotheses and engineer an improved prefusion-stabilized construct if needed.

A recently published study used comparable protein-engineering approaches to stabilize the gB ectodomain into a prefusion-like conformation, named gB-C7. Contrary to our approach, however, this study utilized fewer mutations, specifically the use of two interprotomer disulfides and one cavity-filling substitution [53]. While this stabilized construct preserved some native epitopes of gB, as demonstrated by its ability to bind known mAbs 1G2 and 7H3, its overall appearance was splayed [53] relative to the putative prefusion structure of HCMV gB [24]. While our construct appears to more closely resemble the putative prefusion structure at a low-resolution, we were unable to achieve 100% prefusion stabilization like the aforementioned study. This and our study demonstrate the difficulty in stabilizing HCMV gB in prefusion form but suggest ways for design of improved gB-based antigens.

Our study and previous iterations of prefusion stabilization strategies, collectively, raise an interesting question. Namely, whether the putative prefusion conformation captured by one structure [24], indeed, represents the native prefusion state, or, whether the prefusion form is too dynamic to be accurately represented by a single conformation. It may be possible that the true prefusion state is, instead, a population of multiple closely related conformations. Studies of other viral fusogens, such as the rabies virus glycoprotein (RABV-G) [83] and the SARS-CoV-2 Spike protein [84], have demonstrated that their respective prefusion conformations adopt multiple dynamic states, likely, due to the storage of high energy required to overcome the kinetic barrier to fusion. Therefore, pEW62, gB-C7 [53], the putative prefusion HCMV gB structure [24], and the putative prefusion structures of other gB homologs [61,69,85,86] may all represent closely related prefusion conformations within the conformational continuum of HCMV gB.

It should be noted that the residual presence of the postfusion conformation in pEW62, and pEW21, does pose some limitations in the interpretation of our findings, as well as downstream applications for both constructs. The majority of the mAbs isolated in this study preferentially bound pEW21 or pEW2 despite being isolated with pEW62. While it is unlikely that the minority postfusion species in purified pEW62 was antigenically dominant during mAb isolation, we cannot rule out the possibility that its presence skewed the types of mAbs we were able to isolate. This may also indicate that antigenically, pre- and postfusion conformations of gB are similar. Future mAb isolation or immunization experiments may benefit from using the previously published prefusion-like gB (gB-C7) [53] in tandem with pEW62 to elicit and/or obtain optimal prefusion-specific mAbs. By isolating additional prefusion-specific mAbs, the field can conclusively determine whether their conformational specificity predisposes them to be robust neutralizers, as has been done for HSV-1 [69,85].

Nevertheless, the development of pEW62 and the isolation of prefusion-specific, cross-strain neutralizing antibodies represent a promising step toward HCMV vaccine and therapeutic design. Future studies should aim to further elucidate the structural basis for neutralization by prefusion-specific antibodies, using higher-resolution cryo-EM or crystallography to map these epitope interactions in detail. Additionally, *in vivo* studies evaluating the protective efficacy of these mAbs and their potential for Fc-mediated functions will be crucial to understanding their full therapeutic potential. Given the morbidity associated with HCMV, particularly in immunocompromised individuals and congenital infections, the advancement of prefusion-stabilized gB as an immunogen holds great promise for public health.

## Materials and methods

### Ethics statement

PBMC were collected from adults without HIV who were recruited at the Seattle HIV Vaccine Trials Unit (Seattle, Washington, USA) as part of the study “Establishing Immunologic Assays for Determining HIV-1 Prevention and Control”, also referred to as Seattle Assay Control (SAC) Cohort. All participants signed informed consent, and the Fred Hutchinson Cancer Center (Seattle, Washington, USA) Institutional Review Board approved the SAC protocol (FHIRB0005567) prior to study initiation. Donors were selected based on screening for previous CMV infection but otherwise selected randomly and no considerations were made for age or sex.

All mice used in our studies were housed with free access to food and water with a 12:12 light: dark cycle. The animal facilities are accredited by the Association for Assessment and Accreditation of Laboratory Animal Care. Mice were handled in accordance with the NIH Guide for the Care and Use of Laboratory Animals, and experiments were approved by the Fred Hutch Cancer Center Institutional Animal Care and Use Committee and Institutional Review Boards (PROTO000051021).

### Cells

HEK293-EBNA1-6E cells (293-6E cells) (RRID: CVCL_HF20) were cultured in Freestyle 293 expression medium (Thermo Fisher, Cat# 12338026) and maintained at 37 °C and 5% CO_2_ with gentle shaking at 130 rpm. Human Foreskin Fibroblasts (HFFs) (Hs27, ATCC: CRL-1634) were cultured from frozen and were grown in Dulbecco’s modified Eagle medium (DMEM) containing 10% Fetal Bovine Serum (FBS), 1% Penicillin-Streptomycin and 2 mM L-Glutamine. The culture was put into the 37 ºC, 5% CO_2_ humidified incubator and split at 100% confluence every 48–72 hours. Cells were passaged up to 20 times before a new stock was thawed to ensure optimal experimental conditions.

### Antibodies

Hybridomas producing mouse anti-HCMV-gB 27–156 and 7–17 mAbs were provided by Dr. William Britt (U. Alabama). IgG forms of these mAbs were produced and purified by Cell Essentials. IgG forms of human anti-HCMV-gB 1G2 and SM5–1 mAbs were produced and purified by GenScript.

### Viruses

HCMV strain AD169 that expresses an eGFP transgene from the HCMV major immediate early promoter (MIEP) inserted between US9 and US10 [72] was a gift from Dr. Donald Coen, Harvard Medical School. TRus11GFP (TR) and TBus11GFP (TB40/e) strains, generated from the BAC clones of TB40/e and TR strains by replacing US11 gene with a GFP transgene under the control of an HCMV IE promoter [73], were gifts from Dr. Brent Ryckman (U. of Montana). These viral strains are referred to throughout this study as AD169_GFP_, TR_GFP_, and TB40/e_GFP_.

### Plasmids and cloning

To generate HCMV gB ectodomain constructs for insect cell expression using recombinant baculovirus, mutations were introduced into the pFastBac1::gB_AD169_706-7M (pSS9) construct [25] using specific primers. The resulting constructs contain an endogenous N-terminal honeybee melittin signal peptide, 7M mutations in the fusion loops, and a C-terminal His_6_ tag (S1 Table). The plasmid sequences were confirmed by Sanger sequencing.

To generate HCMV gB ectodomain constructs for mammalian expression, cDNA corresponding to HCMV gB_AD169_ in constructs pEW2_Sf9_, pEW21_Sf9_, and pEW62_Sf9_ were amplified by PCR using Platinum SuperFi II DNA polymerase Master Mix (Invitrogen, Cat# 12368050), and gene specific primers according to the manufacturer’s instructions and subcloned in frame into a pTT3 plasmid. The resulting constructs pEW2, pEW21, pEW62, contain N-terminal TPA signal peptide and a C-terminal HisAvi tag. The plasmid sequences were confirmed by Sanger sequencing.

### HCMV gB production

Plasmid DNA encoding pEW62-HisAvi, pEW21-HisAvi, or pEW2-HisAvi was mixed with PEI MAX (Polysciences, Cat# 24765) at a 3:1 ratio in 1X PBS and incubated for 20 min at room temperature before dropwise addition to 293-6E cells at 1 x 10^6^ cells/ mL in Freestyle media. The transfected culture was incubated at 37 °C shaking at 130 rpm with 5% CO_2_ for six days. On day six, the culture was centrifuged at 4000 x *g* for 10 minutes at 4 °C. The supernatant was collected and adjusted to 500 mM NaCl, 10 mM Imidazole, 0.02% sodium azide and then clarified through a 0.22-µm filter (Corning, Cat# 431097). The clarified supernatant was then passed over Ni-NTA resin (Gold Biotechnology, Cat# H-350–100) pre-equilibrated with Ni-NTA binding buffer (10 mM Tris, 500 mM NaCl, 10 mM imidazole, 0.02% sodium azide, pH 7.1), followed by extensive washing with Ni-NTA binding buffer, and then eluted with Ni-NTA elution buffer (10 mM Tris, 500 mM NaCl, 500 mM imidazole, 0.02% sodium azide, pH 8.0). Ni-NTA captured proteins were concentrated using 30 kDa centrifugal filter (Millipore, Cat# UFC9030) and purified by size-exclusion chromatography (SEC) using a HiLoad 16/600 Superdex 200 pg column (Cytiva, Cat# 28989335) in 1X PBS running buffer. pEW62-HisAvi was then further purified via HiLoad 16/600 Superose 6 pg column (Cytiva, Cat# 29323952).

### Antibody production

Plasmids encoding antibody heavy and light chains were combined with PEI Max at a 3:1 ratio in PBS and incubated at room temperature for 20 minutes. The plasmid mixture was added to 293-6E cells at 1 x 10^6^ cell/ mL in Freestyle media (Thermo Fisher, Cat# 12338026) and incubated at 37 °C shaking at 130 rpm with 5% CO_2_ for six days. On day six, the cultures were centrifuged at 4000 X *g*. The supernatant was passed through a 0.45-µm Steriflip filter (Millipore, Cat# SE1M003M00). The supernatant was passed over protein A agarose resin (Gold Biotechnology, Cat# P-400) pre-equilibrated with 1X PBS. The resin was then washed with 5 column volumes of 1X PBS. The antibody was then eluted off the resin using IgG elution buffer (Pierce, Cat# 21004). It was eluted 1 mL at a time into microcentrifuge tubes containing 100 µL of 1M Tris pH 8.0. The harvested antibody was buffer exchanged into 1X PBS using 30 kDa centrifugal filter (Millipore, Cat# UFC9030) and stored at 4 °C.

### Fab production and digestion

To generate VH-Fab plasmid, cDNA corresponding to the VDJ regions of MLCB1-, MLCB6-, MLCB7-, MLCB9-, MLCB10-, and MLCB11- was amplified with Platinum SuperFi II DNA polymerase (Invitrogen, Cat# 12368050) and cloned in frame with an expression vector encoding the IgG constant region 1 followed by a HisAvi-tag. DNA was prepared using QIAprep Spin Miniprep Kit (Qiagen, Cat# 27106) and Sanger Sequenced.

To generate MLCB1-, MLCB6-, MLCB7-, MLCB9-, MLCB10-, and MLCB11-Fab, corresponding paired VH-Fab and VL plasmids were co-transfected in 293-6E cells using PEI Max at a 3:1 ratio. Fabs were harvested from supernatants using Ni-NTA Agarose following the same procedures described under “Expression and purification of CMV gB variants” above.

MLCB5-Fab was generated by digesting MLCB5 antibody with LysC endoproteinase enzyme (NEB, Cat# P8109S) in 2000:1 ratio at 37 °C for overnight. The digested lysate was then incubated with protein A at RT for 120 minutes to capture Fc fragment and undigested IgG. Flow through containing the MLCB5-Fab was, concentrated using a 30-kDa centrifugal filter and further purified by size exclusion chromatography with HiLoad 16/600 Superdex 200 pg column (Cytiva, Cat# 28989335) using 1X PBS running buffer.

To generate 1G2- and SM5–1-Fab, the corresponding antibody was digested with papain enzyme using the Pierce Fab Micro Preparation Kit (Thermo Scientific, Cat# 44985). The digested lysate was then incubated with protein A&G (Thermo Scientific, Cat# 89958) at RT for 1 hour to capture Fc fragment and undigested IgG. Flow through containing the 1G2- or SM5–1-Fab was concentrated using a 30 kDa centrifugal filter. Digestion and purification were verified by 12% acrylamide gel and Coomassie blue stain.

### Biotinylation of HisAvi-tagged gB variants

The CMV gB variant proteins were biotinylated with BirA (Avidity LLC, Cat# BirA500) according to the manufacturer’s instructions. Excess biotin was removed using Zeba spin desalting columns (Thermo Fisher, Cat# 87766) and used immediately or stored at -80 °C after flash-freezing.

### Fluorescently labeled streptavidin tetramer production

Biotinylated pEW62 was conjugated to either streptavidin-R-phycoerythrin (SA-PE, Agilent, Cat# PJRS301-1) or streptavidin allophycocyanin (SA-APC, Agilent, Cat# PJ27S-1) at 4:1 molar ratio. A PE-decoy reagent was produced by conjugating SA-PE to DyLight 650 NHS Ester-(DL650;Thermo Fisher, Cat# 62266) by mixing 1 mg Agilent SA-PE into one vial of 50 µg DL650 and incubating at RT for 60 minutes. Excess dye was removed with 2 mL Zeba spin desalting column (Thermo Fisher, Cat# A57761) and used immediately or stored at 4 °C in an opaque black microcentrifuge tube. SA-PE-DL60 was incubated with irrelevant Avi-tagged biotinylated protein from *Plasmodium yoelii* (PY-gamma), a kind gift from Dr. D.N. Sather [70], at a 4:1 SA to PY-gamma ratio. Proteins and SA conjugates were incubated in the dark at RT for 5 minutes followed by an addition of 25 nmol D-biotin (Invitrogen, Cat# B20656) to quench unoccupied binding sites on SA.

### Antigen-specific B cell sorting

4 x 10^7^ human PBMCs from CMV seropositive donors were thawed in 2 mL of heat-inactivated (HI) FBS (Sigma-Aldrich, Cat# F0926). Then, the volume was adjusted to 25 mL with complete RPMI (Corning, Cat# 15–040-CV containing 10% HI-FBS, 1% Penicillin-Streptomycin (Gibco, Cat# 15140122), and 1% L-Glutamine (Gibco, Cat# 25030081)) (cRMPI) followed by centrifugation at 500 x *g* for 3 minutes at RT. B cells were enriched by negative selection using the EasySep Human B Cell Isolation kit (STEMCELL Technologies, Cat# 19054) according to the manufacturer’s instructions. The enriched sample was incubated in 100 µL of FACS buffer (1X PBS, 2% HI-FBS, and 1mM EDTA) containing rat serum (STEMCELL Technologies, Cat# 13551), mouse serum (Sigma-Aldrich, Cat# M5905), mouse-anti-human-CD32 (BD Biosciences, Cat# 551900), mouse-anti-human-CD23 (BD Biosciences, Cat# 555707), and mouse-anti-human-CD16 (BD Biosciences Cat# 550383) all at 1:200 dilution, for 15 minutes at 4 °C. Cells were washed with 500 µL of FACS buffer then stained with 1 µM of pEW62 conjugated to SA-PE, 1 µM of pEW62 conjugated to SA-APC, 1 µM of PY-gamma conjugated to SA-PE-DL65, anti-CD3-FITC (BD Biosciences, Cat# 556611) at a 1:100 dilution, anti-CD14-FITC (BD Biosciences, Cat# 557153) at a 1:100 dilution, anti-IgD-PerCP-Cy5.5 (eBioscience, Cat# 46-9868-42) at a 1:100 dilution, anti-IgG-BV421 (BD Biosciences, Cat# 561299) at a 1:200 dilution, anti-CD19-BV711 (BioLegend, Cat# 302246) at a 1:200 dilution, and a fixable viability dye eFluor 506 (eBioscience, Cat# 65-0866-14) at a 1:100 dilution in 100 µL of FACS buffer. The cells were incubated in the dark at 4 °C for 30 minutes followed by a wash and resuspended in 500 µL of FACS buffer at 4 °C until sorting. Live, antigen-positive, and class-switched B cells (CD3^-^, CD14^-^, Live/dead^-^, CD19+ , IgG+ , IgD^-^, PE-DL650^-^, PE^+^, APC+ ) were singly sorted into 96-well plates using BD FACSymphony S6. The cells were frozen on dry ice and stored at -80°C.

### VH/VL transcript amplification and cloning

cDNA was generated using Superscript IV Reverse Transcriptase (Invitrogen, Cat# 18090200) in a 20 µL reaction according to the manufacturer’s instructions. VH and VL transcripts were amplified using nested PCR. The first-round reaction contained 5 µL of cDNA, 1 unit of HotStarTaq Plus DNA polymerase (Qiagen Cat# 203605), 250 nM of 3′ primer pool and 380 nM of 5′ primer pool [87], 25 mM of dNTP (Invitrogen, Cat# 10297018), 2 µl of 10X buffer, and 12.4 µL of nuclease-free H_2_O. Second round PCR reaction used 5 µL of first round PCR product as template and 250 nM of both 5′ and 3′ primers. Both reactions were cycled at 94 °C for 5 minutes, 50 cycles of 94 °C for 30 sec, 57 °C (IgG and IgK) or 60 °C (IgL) for 30 sec, and 72 °C for 55 sec, followed by 72 °C for 10 minutes. Second round PCR primers included homology regions that correspond to the leader sequence and constant regions on the expression vector [88]. Second round PCR products were subject to electrophoresis on a 1% agarose gel containing 0.1% GelRed nucleic acid gel stain (Gold Biotechnology, Cat# G-725–500). 10 μL of reactions corresponding to wells with positive amplicons were purified using Monarch PCR & DNA Cleanup Kit (NEB Cat# T1030L) and eluted in 8 μL DNA Elution Buffer. Amplicons were cloned directly into heavy and light chain expression plasmids [88] using In-Fusion HD Enzyme (Takara Bio Cat# 639650) according to the manufacturer’s instructions. Expression plasmids were confirmed by Sanger Sequencing. Recombinant mAbs were expressed and purified as described under “Antibody Production”.

### Antibody binding by Biolayer Interferometry (BLI)

All experiments were carried out on an Octet RED96e at 30 °C with 1000 rpm shaking.

#### Initial binding screens.

mAbs were diluted to 15 µg/mL in 1X PBS containing 0.01% BSA, 0.02% Tween-20, and 0.005% sodium azide (Kinetics Buffer or KB) and captured on anti-human IgG Fc Capture (AHC) biosensors (Sartorius, Cat# 18–5063) for 100 sec. Sensors were immersed in KB for 60 sec to collect a baseline measurement. The sensors were then immersed in KB containing 40 µg/mL of pEW62-HisAvi, pEW21-HisAvi, or pEW2-HisAvi for 150 sec to measure association. The maximum binding response is defined as the nm shift at 150 s. All measurements of antibody binding were corrected by subtracting the signal obtained from incubating AHC sensors without mAb in KB containing the gB proteins.

#### Competition BLI.

mAbs were biotinylated at 1:1 molar ratio with EZ-Link NHS-PEG4 biotin (Thermo Fisher, Cat# A39259), and excess biotin was removed using a Zeba spin desalting column. Biotinylated mAbs were loaded onto streptavidin (SA) biosensors (Sartorius, Cat# 18–5019) (at 15 µg/mL) for 100 sec followed by a baseline reading in 1X PBS containing 0.01% BSA, 0.02% Tween-20, and 0.005% sodium azide (Kinetics Buffer or KB) for 60 sec. mAb-loaded sensors were then immersed in wells containing 50 nM of pEW62-HisAvi alone or, 50 nM of pEW62-HisAvi that had been pre-incubated with 133.5 nM of non-biotinylated mAb for 60 minutes. Association was measured for 150 sec, and the sensors were then immersed in KB to measure dissociation for 500 sec.

An additional well containing KB for association was used as internal control for background subtraction. Relative binding was calculated by taking maximum binding responses (R_max_) of the biotinylated mAb to the mAb/pEW62 complex measured during the association phase divided by R_max_ of the biotinylated mAb to pEW62-HisAvi in the absence of mAb and then multiplied by 100%. Binding inhibition was calculated by subtracting the relative binding value from 100.

#### Binding kinetics.

To measure affinity, biotinylated pEW62, pEW21, or pEW2 was immobilized on streptavidin (SA) biosensors for 150 sec and then immersed in KB for 30 sec to collect a baseline. Sensors were then immersed in wells containing two-fold serial dilutions of Fabs in KB buffer and the association was measured for 300 sec. The sensors were then immersed in KB for 600 sec to measure dissociation. The background signal from each analyte-containing well was measured using empty reference sensors and subtracted from the signal obtained with each corresponding ligand-coupled sensor at each time-point. Kinetic analyses were performed at least twice with an independently prepared analyte dilution series. Curve fitting was performed using a 1:1 binding model in Octet BLI Analysis 12.2 software. Mean k_dis_ (1/s) and k_on_ (1/Ms) values were determined by averaging all binding curves that matched the theoretical fit with an R^2^ value of ≥ 0.95. The K_D_ value of each neutralizing Fab versus each gB molecule was calculated by taking the mean k_dis_ value divided by the mean k_on_ value.

### Western blotting

0.1 µg of pEW62-HisAvi alongside 5 µL of pre-stained protein ladder (Thermo Scientific, Cat# 26619) was separated by SDS-PAGE using premade bis-tris 4–12% protein gel (Invitrogen, Cat# NP0321BOX) under reducing conditions according to the manufacturer’s instructions. Protein was transferred to PVDF membrane (Invitrogen, Cat# IB401002) with an iBlot 2 gel transfer device (Invitrogen, Cat# IB21001) following manufacturer’s instructions. The membrane was then incubated in blocking buffer (10% nonfat dry milk (Apex Bioresearch, Cat# 20–241) and 0.1% Tween-20 in 1X PBS) at room temperature while shaking at 250 rpm for 60 minutes. The membrane was cut into five strips with lanes containing pEW62-HisAvi alongside the protein ladder, and each strip was blotted with an individual mAb at 0.2 µg/mL in 10 mL of blocking buffer. The blots were incubated at 4 °C shaking at 250 rpm overnight. The blotted PVDF strips were rinsed in wash buffer (0.1% Tween-20 in 1X PBS) three times followed by three washes in wash buffer at room temperature for 5 minutes. Each strip was then incubated with either anti-human IgG-HRP antibody (Southern Biotech, Cat# 2010-05) at 1:3000 or anti-mouse IgG-HRP antibody (Southern Biotech, Cat# 1033-05) at 1:5000 in 10 mL of blocking buffer. The blots were incubated at RT with shaking at 250 rpm for 60 minutes followed by three quick rinses in wash buffer and three 5 minutes washes in wash buffer. The dabbed dried blots were incubated with WesternBright Quantum substrate (Advansta Inc, Cat# K-12042-C20) and incubated for 2 minutes. The signals were captured through Bio-Rad Gel-Doc System for 0.2 sec of exposure time under colorimetric setting, and either 0.2 sec or 0.5 sec under chemiluminescent setting. The images were overlaid for data analyses using Bio-Rad Image Lab 6.0.

### Permeabilized cell staining and cell surface staining

pcDNA-HCMV-AD169 FL gB plasmid was transfected into 293-6E cells at 1 X 10^6^ cells/mL following the manufacturer’s instructions and incubated at 37 °C with 5% CO_2_ shaking at 130 rpm. 24 hours later, untransfected and transfected cells were centrifuged at 500 x *g* for 3 minutes at room temperature followed by a 1X PBS wash. 8 X 10^6^ cells were stained in 2 mL of 1X PBS containing fixable viability dye eFluor 506 (eBioscience, Cat# 65-0866-14) at a 1:200 dilution. Cells were incubated at RT for 20 minutes in the dark followed by centrifugation and a 1X PBS wash. Untransfected and transfected cells were subsequently divided into two for intracellular and cell surface staining respectively.

To perform permeabilized cell staining, 4 X 10^6^ cells were fixed in 4 mL of 1X IC fixation buffer (eBioscience, Cat# 88–8824) and incubated at room temperature for 20 minutes in the dark. The cells were then permeabilized in 4 mL of 1X permeabilization buffer (eBioscience, Cat# 88–8824) and incubated at room temperature for 5 minutes. At the end of the incubation, cells were dispensed into a 96-well plate at a density of 2 X 10^5^ cells/well followed by centrifugation. 100 µL of 1X permeabilization buffer containing 0.5 µg/mL of each mAb was added to transfected or non-transfected permeabilized cells. One well containing the anti-EBV mAb, AMMO4, was used as an isotype control, and one well containing buffer alone was used as a no mAb control. Cells were incubated at RT for 30 minutes followed by centrifugation and a 1X permeabilization buffer wash. Each well was then resuspended in 100 µL of 1X permeabilization buffer containing PE-goat-anti-human IgG (Jackson ImmunoResearch, Cat# 109-117-008) at 1:50. Cells were incubated at RT for 30 minutes in the dark followed by centrifugation and a 1X permeabilization buffer wash. Cells were resuspended in 200 µL of FACS buffer (1X PBS, 2% heat inactivated-FBS, and 1mM EDTA).

To perform cell surface staining, 4 X 10^6^ transfected and 4 X 10^6^ untransfected cells were independently resuspended in 4 mL of FACS buffer and dispensed into 96-well plates at a density of 2 X 10^5^ cells/well followed by centrifugation. Cells were resuspended in 100 µL of FACS buffer containing 0.5 µg/mL of each isolated mAb and incubated on ice for 1 hour. The cells were centrifuged and washed with FACS buffer followed by addition of 100 µL of FACS buffer containing PE-goat-anti-human IgG at 1:50 and incubated for 1 hour on ice in the dark. The cells were centrifuged and washed with FACS buffer and fixed in 200 µL of 10% formalin on ice for 20 minutes. The cells were centrifuged and washed with FACS buffer one more time before being resuspended in 200 µL of FACS buffer.

Stained cells were analyzed on BD X50 and human-IgG-positive live cells (Live/dead- and PE+) were recorded. The percentage of each mAb-positive (human-IgG-positive) cell population among all the live cells was plotted in GraphPad Prism 10.2.3.

### Infected cell staining

Human Foreskin Fibroblasts (HFFs) (Hs27, ATCC: CRL-1634) were seeded in 6-well plates at a density of 3 × 10^5^ cells per well. The cells were incubated at 37 ºC for 3 days to ensure 100% confluency. On the third day, cells were washed with 1X PBS and then infected with HCMV-AD169_GFP_ at an MOI of 3 in 500 μL of culture media per well (DMEM containing 10% FBS, 1% P/S, and 2mM L-Glutamine) for 1 hour. The plate was rotated every 15 minutes to promote even distribution of the infection. After the incubation, the media containing the virus was aspirated, the cells were rinsed with media, and 2 mL of fresh media was added to each well. The cells were returned to 37 ºC, 5% CO_2_. One well was left uninfected as a control.

18 hours post-infection, the cells were washed with pre-warmed 1X PBS and treated with 500 μL of pre-warmed Versene (Gibco, Cat. #15040066) for 10 minutes. Following incubation, the cells were removed from the wells by gentle pipetting into 1.5 mL Eppendorf tubes, and each well was washed with 500 μL of FACS Buffer (1X PBS, 3% FBS). The cells were centrifuged and washed with 2X with FACS Buffer. They were then stained with 0.5 μg/mL of primary mAb (MLCB mAbs, 1G2, or SM5–1) for 1 hour at 4 ºC. Infected and uninfected cells with no primary mAb were used as controls. Cells were centrifuged, washed 3X with FACS Buffer, and stained with PE-goat-anti-human IgG (Jackson ImmunoResearch, Cat# 109-117-008) at 1:250 for 1 hour at 4 ºC. Cells stained with secondary mAb alone were used as controls. Following staining, cells were centrifuged, washed 2X with FACS Buffer, and fixed with 4% PFA for 10 minutes. Following fixation, the cells were centrifuged, washed 2X with FACS Buffer, resuspended in FACS Buffer, and transferred to 5 mL round-bottom tubes for analysis on a Cytek Aurora for GFP and PE signals.

The percentage of each mAb-positive (human-IgG-positive) cell population among all the infected (GFP+) cells was quantified in FlowJo Software, v10 and plotted in GraphPad Prism 10.2.3. Full gating strategy can be found in S5 Fig.

### Immunizations

8 to 9-week-old C57BL/6 male and female mice (The Jackson Laboratory, Strain# 000664) were randomly assigned into three groups of five and immunized with 10 ug of pEW62, pEW21, or pEW2 in 1X PBS adjuvanted with 50 µL of PBS-reconstituted Sigma Adjuvant System (SAS) (Sigma-Aldrich, Cat# S6322-1VL) in a total of 100 µL per dose. Immunizations were delivered on weeks 0, 4, and 8 by intramuscular (IM) injection split between both hind legs (50uL each leg). Bleeds were collected retro-orbitally (RO) at weeks 0, 2, 4, 6, and 8. Terminal bleed was collected through cardiac puncture at week 10.

### ELISA on immunized mouse sera

384-well clear flat-bottom plates (Thermo Fisher, Cat# 464718) were coated overnight with 60 ng/well of either pEW62, pEW21, or pEW2 in 30 µL of 0.1 M sodium bicarbonate. The next day, the wells were washed three times with 1X PBS containing 0.02% Tween-20 (wash buffer) and then blocked with 100 µL of 1X PBS containing 10% nonfat dry milk and 0.03% Tween-20 (dilution buffer) and incubated at 37 °C for 90 minutes. The plates were washed three times with 1X wash buffer. 45 µL of dilution buffer containing three-fold serial dilutions of mouse sera were added in duplicate. 24 wells were incubated with 30 µL of dilution buffer alone as a background control. The plates were incubated at 37 °C for 60 minutes followed by three washes with 1X wash buffer. The wells were incubated with 3 µL of dilution buffer containing anti-mouse IgG-HRP antibody (Southern Biotech, Cat# 1033-05) at 1:5000 for 60 minutes at 37 °C. The plates were washed three times with 1X wash buffer. 30 µL of pre-warmed (37 °C) TMB Peroxidase Substrate (SeraCare, Cat# 53-00-02) was added into each well and developed at room temperature for 5 minutes followed by the addition of 30 µL of 1N sulfuric acid to stop the reaction. The absorbance at 450 nm was measured using a Molecular Devices SpectraMax i3x microplate reader.

The A_450_ values from the control wells were averaged and subtracted from that of each serum containing well and plotted against the serum dilution. Nonlinear regression using the Sigmoidal, dose-response (variable slope) least squares fit function in the GraphPad Prism 10.2.3 package was used to determine the half maximal binding (EC_50_) for each serum sample. Statistical differences between groups were at each timepoint were determined using a Mann-Whitney test in the GraphPad Prism 10.2.3 package.

### Virus propagation

All HCMV strains used in this study encoded a GFP transgene under the control of an IE promoter; see “Viruses” for genetic details and origins. All stocks (P0) were expanded to generate P1 stocks. The P1 stocks were then used to create P2 working stocks, which were used for all experiments performed in this study. All working stocks were made from original P1 stocks to limit passages in HFFs and mitigate potential mutations.

To generate P1 and P2 stocks, HFFs were seeded in a T175 flask 48 hours before starting the virus propagation to ensure 100% confluency during infection. Once they reached 100% confluency, the virus was added at an MOI of 0.01 in 5 ml of culture media (DMEM containing 10% FBS, 1% P/S and 2mM L-Glutamine) for 1 hour. The flask was rotated every 15 minutes to promote even distribution of the infection. After the incubation, the media containing the virus was aspirated, the cells were rinsed with media, and 25 mL of fresh media was added to the flask.

The flask was incubated for 14 days (AD169_GFP_) or 21 days (TR_GFP_ and TB40/e_GFP_) at 37°C and examined under the microscope for the number of infected cells (GFP-positive) every 4 days. Once 100% of cells were infected, the cells and media were collected, sonicated, and centrifuged to remove cellular debris. The supernatant containing the virus was aliquoted and placed directly into liquid nitrogen, then transferred to a -80 ºC freezer until it was used. Once thawed, virus stocks were used or discarded and were not refrozen for subsequent use.

### Virus titration

Viral stocks were titrated by using a 50% tissue culture infectious dose (TCID_50_) assay [89] shared by Dr. Felicia Goodrum (University of Arizona). HFFs were seeded on a 96-well plate at 1 x 10^4^ cells/well and incubated at 37 ºC overnight, to allow them to adhere. The virus was then serially diluted from 10^-1^ to 10^-8^. 100 µL of each aliquot was added to the plate with 12 replicates each (i.e., one dilution per row). The plate was incubated at 37 ºC for 14 days. The wells were then washed twice with cold PBS and fixed with 4% paraformaldehyde (PFA) in the dark for 10 minutes. PFA was then aspirated, and the plate was washed two times with PBS and left in PBS. The cells were then visually assessed for the presence of GFP signal. A well was considered to be GFP positive if two or more cells in the well were GFP positive. The total number of GFP-positive wells in each row was recorded and then converted to an approximate titer value.

### Neutralization assays

HFFs were seeded on a 96-well plate at 1 x 10^4^ cells/well. The cells were incubated at 37 ºC for 3 days to ensure 100% confluency. Antibody dilutions were prepared in cell culture media (DMEM containing 10% FBS, 1% Pen/Strep, and 2 mM L-Glutamine) with the following method: first, a 2X stock of each desired antibody concentration and a 2X virus stock, were made; secondly, the 2X antibody and virus stocks were mixed 1:1 to have a 1X concentration of both. For AD169_GFP_, the final MOI was 30, for TR_GFP_ and TB40/e_GFP,_ it was 5. TR_GFP_ and TB40/e_GFP_ do not grow to sufficiently high titers to achieve an MOI of 30 within the volume confines of this assay, so the assay had to be adjusted to account for a lower experimental MOI at the same volume. We used equivalent GFP RFU values to determine comparable MOI and timepoints for each strain (18 hours vs. 48 hours). Then, the mixtures were pre-incubated at 37 ºC for 2 hours. After the pre-incubation, the media from the wells was aspirated, and 100 μL of each mixture was added to a 96-well plate in triplicate.

For mouse sera neutralization assays, a similar approach was used. Sera were diluted to a starting concentration of 1:25 (2X stock) and serially diluted 1:4. For the positive control, known nAb 14-4b [67] was added to naïve mouse sera at 200 μg/mL (2X stock) and diluted with the sera 1:4. They were then mixed with a 2X stock of AD169_GFP_ 1:1 (final MOI of 30) and pre-incubated at 37 ºC for 2 hours. After the pre-incubation, the media from the wells was aspirated and 100 μL of each mixture was added to the corresponding wells in duplicate. Serum from each mouse, n = 7 (pEW62-immunization group), n = 5 (pEW21-immunization group, pEW2-immunization group), and n = 3 (naïve mice, 14-4b) was tested individually in technical duplicate. The mean percent neutralization of each immunization group was calculated in GraphPad Prism 10.2.3 and plotted.

Once the antibodies and virus were added to the 96-well plate, it was incubated for 18 hours (AD169_GFP_) or 48 hours (TR_GFP_ and TB40/e_GFP_) at 37 ºC. To maintain adequate GFP RFUs with a lower MOI, the timepoint was extended to 48 hours from 18 hours. The wells were washed twice with cold PBS, and after aspiration, 4% paraformaldehyde (PFA) was added to the wells to fix the cells. The plate was then incubated at room temperature in the dark for 10 minutes. When the PFA was aspirated, the plate was washed twice with PBS and left in PBS until it was read via a fluorescent plate reader (BioTek H1) for bulk fluorescent signal.

### Calculation of nAb IC_50_ and Standard Error of the Mean (SEM)

To calculate the IC_50_ of the antibodies studied, three biological replicates were performed, all in technical triplicates. After measuring the relative fluorescent units (RFU), the percentage of inhibition was calculated as neutralization (%)=(*MAX − VALUE*)/(*MAX − MIN*)×100, where MAX is the mean of “no mAb” or “virus alone” wells, and MIN is the mean of the negative control (uninfected) wells.

The neutralization (%) was plotted as a function of the antibody concentration using an XY graph on GraphPad Prism 10.2.3. The concentrations were transformed to the logarithm of the concentration and graphed as a log(inhibitor) vs. response – variable slope (four parameters) curve. Finally, the half-maximal inhibitory concentration (IC_50_) was interpolated on this curve and the antilogarithm was calculated.

The IC_50_ values were graphed using GraphPad Prism 10.2.3 in a column chart where the X-axis corresponded to the antibody and the Y-axis corresponded to the IC_50_ values. The statistical description of this chart was provided by the software to determine the SEM.

### Negative-stain electron microscopy

#### Sample preparation.

pEW62, pEW21, and pEW2 were purified as described in “HCMV gB production” above and aliquoted into small volume working stocks, stored at -80 °C. Stocks were thawed on ice for 20 minutes, and protein concentration was measured using a NanoDrop. They were diluted to 5 μg/mL in 1X PBS buffer and immediately applied to glow-discharged copper/carbon film grids (EMS, Cat# CF400-CU-50) for 30 seconds and blotted with Wattman paper. The grid was then washed twice with dH_2_O with blotting on Wattman paper in between washes. The grid was then stained with 0.075% uranyl formate for 60 seconds before blotting on Wattman paper. Micrographs were collected on a Tecnai G2 Spirit BioTwin transmission electron microscope at 120 kV and 49,000 x magnification (1.43 Å/px) and processed in CryoSPARC [90].

Purified pEW62 and purified Fab were thawed on ice for 20 minutes. They were then mixed at a 2-fold molar excess of Fab to gB monomer (in 1X PBS buffer) to a solution with a final volume of 250 µL and incubated on ice overnight. The samples were then run through a Superose 6 10/300 GL column (Cytiva, Cat# 29091596). The eluted fractions were analyzed by SDS–PAGE followed by Coomassie blue staining. The fractions containing the complex were pooled and concentrated using Millipore Amicon 50 kDa MWCO centrifugal filter (Sigma Aldrich, Cat# UFC8050). The concentration of the complex was measured using NanoDrop and then stored at 4 °C overnight. It was then cross-linked with 0.025% Glutaraldehyde for 10 minutes at RT and quenched with 36 mM Tris-HCl, pH 8.0. The complex was then diluted to 5 µg/mL and immediately applied to glow-discharged copper/carbon film grids (EMS, Cat# CF400-CU-50) for 30 seconds and blotted with Wattman paper. The grid was then washed twice with dH_2_O with blotting on Wattman paper in between washes. The grid was then stained with 0.075% uranyl formate for 60 seconds before blotting on Wattman paper. Micrographs were collected on a Tecnai G2 Spirit BioTwin transmission electron microscope at 120 kV and 49,000 x magnification (1.43 Å/px) and processed in CryoSPARC [90].

#### Data processing.

A minimum of 100 micrographs per sample were collected and imported into CryoSPARC [90]. All micrographs collected were used for data processing. Particles were selected using the blob picker feature, with a particle radius of 100 Å – 200 Å for pEW62, pEW21, and pEW2. These measurements were taken from the study by Liu et al. For pEW62/Fab complexes, a particle radius of 150 Å – 200 Å was used to account for the extra particle width as a result of the Fab(s). All picked particles were extracted with a box size of 400 px and 2D classed with one class per 1,000 particles (i.e., a dataset with 50,000 particles would be sorted into 50 2D classes) with force max over poses turned off, enforcing non-negativity turned on, clamp-solvent turned on, and no CTF correction. All 2D classes that represented any distinct particle shape (i.e., not noise or trash) were subsequently selected and re-classified into a smaller number of classes with similar parameters as previously described. Once all noise and “trash” classes were filtered out, particles were manually visually quantified and grouped into different conformations for pEW62, pEW21, and pEW2. After manual quantification, all particles were classed into 6–10 ab-initio volumes. In the case of pEW62/Fab complexes, visually similar volumes were grouped by homogenous refinement to produce the final 3D reconstructions. In the case of pEW62, pEW21, and pEW2, visually different volumes (i.e., conformations) were further refined by heterogeneous refinement. For pEW62, pEW21, and pEW2, C3 symmetry was applied after the heterogeneous refinement step. No symmetry (C1) was applied to the pEW62/Fab complexes, as many only showed one or two Fabs bound. Final volumes were exported into ChimeraX for model generation.

#### Generation of gB/Fab models.

To obtain a model of the prefusion gB ectodomain bound to SM5–1 Fab, first, the structure of HCMV gB dII bound to SM5–1 L_V_H_V_ (extracted from RCSB PDB 7KDD) was overlaid onto the structure of the prefusion gB ectodomain (extracted from RCSB PDB 7KDP) by aligning respective dII domains in PyMOL. This resulted in the model of the prefusion gB ectodomain bound to SM5–1 L_V_H_V_. Next, the structure of the full-length SM5–1 Fab (PDB RCSB 4OSU) was overlaid onto the model of the prefusion gB ectodomain bound to SM5–1 L_V_H_V_ by aligning the respective L_V_H_V_ portions in PyMOL. The resulting model contains the prefusion gB ectodomain (extracted from RCSB PDB 7KDP) and the SM5–1 Fab (PDB RCSB 4OSU). This model was then fit into the corresponding NS-EM reconstruction in ChimeraX.

To obtain a model of the prefusion gB ectodomain bound to 1G2 Fab, first, the structure of HCMV gB dI bound to 1G2 L_V_H_V_ (extracted from RCSB PDB 5C6T) was overlaid onto the structure of the prefusion gB ectodomain (extracted from RCSB PDB 7KDP) by aligning respective dI domains in PyMOL. This resulted in a model of the prefusion gB ectodomain bound to 1G2 L_V_H_V_. Next, the L_C_H_C_ extracted from RCSB PDB 7FAB was stitched to the 1G2 L_V_H_V_ in PyMOL. The resulting model contains the prefusion gB ectodomain (extracted from RCSB PDB 7KDP), the 1G2 L_V_H_V_ (PDB RCSB 5C6T), and the L_C_H_C_ extracted from RCSB PDB 7FAB. The model was further verified by aligning it onto the structure of HCMV gB dI bound to 1G2 Fab (extracted from RCSB PDB 8VYN). The dI/1G2 Fab portion of the assembled model was virtually indistinguishable from the structure. This model was then fit into the corresponding NS-EM reconstruction in ChimeraX.

To obtain models of MLCB Fabs, their corresponding L_V_H_V_ sequences were imported into ROSIE to generate L_V_H_V_ models. These L_V_H_V_ models were then stitched with the L_C_H_C_ extracted from RCSB PDB 7FAB, similarly to how it was done for 1G2 Fab. For each pEW62/Fab complex, first, the structure of the prefusion gB ectodomain (extracted from RCSB PDB 7KDP) was manually fitted into the NS-EM reconstruction in ChimeraX. Then, the corresponding Fabs were fitted into the remaining density. In the case of MLCB7, the NS-EM density only allowed for fitting of the MLCB7 L_V_H_V_.

#### Determination of gB orientation in NS-EM map fits.

Due to its shape, pEW62 appeared to have x-axis symmetry in a number of NS-EM maps generated in this study. To ensure accurate classification of binding domains in pEW62/Fab complexes, secondary metrics were used to guide manual fitting into NS-EM maps. For SM5–1 and 1G2, the epitope locations and models were generated from known structures (see “Generation of gB/Fab models” above). Thus, these models lacked ambiguity and were fitted to NS-EM maps accordingly.

In the case of MLCB9 and MLCB11, these mAbs compete with 1G2 and SM5–1; their binding domains were fitted accordingly. In the case of MLCB5, MLCB6, and MLCB7, these were chosen as having Domain I epitopes due to their competition and the unambiguous symmetry of the pEW62/MLCB6 NS-EM map, which did not allow any other orientation besides the one displayed in Fig 7. In the case of MLCB1, this map also did not allow for any other orientation fit besides the one displayed in Fig 7, it was classified as having a Domain II epitope. In the case of MLCB10, the map’s symmetry did not allow us to conclusively determine which domain of gB interacted with the Fab. Thus, both orientations were included in Fig 7.

## Supporting information

S1 Fig Purity and antigenicity of gB variants. (A) purified pEW62, pEW21 and pEW2 were subjected to size exclusion chromatography on an Enrich650 column and the chromatograms are overlaid as indicated. (B) pEW62, pEW21 and pEW2 were separated by SDS-PAGE reducing and non-reducing conditions and then stained with Coomassie blue. MWM = molecular weight marker. (C-F) Additional 2D and 3D classes of gB constructs. Each set of 2D classes for each construct is taken from the same 2D classification “job” in CryoSPARC and includes particles used in generating ab-initio reconstructions (see M&Ms for detailed processing strategy). Trash particles are not shown. Scale for each row of images is indicated on the left-hand side of the first image. (C) All particles are classified as postfusion. (D) Particles classified as intermediate are boxed in gray, corresponding to coloring in Fig 2E and 2F. Postfusion particles are unboxed. These particles have different dimensions than pre- or postfusion, they are much shorter. (E) Additional 3D classes of pEW21 with a cartoon schematic of the hypothesized conformations they represent. From left to right, particle numbers comprising each class are as follows: 5116, 2929, 2981, and 5071. In the case of the first class, it is an average of multiple intermediate particles that have one or more dI “arms” swinging upwards. Flexible regions of particles are not included in averages, as they are more challenging to capture. Thus, the ends of dI, which vary between particles, are not in the average. The same explanation can be used for the other classes. (F) Particles classified as “prefusion” are boxed in purple, corresponding to coloring in Fig 2H and 2I. Postfusion particles are unboxed. (G) The affinity of 1G2 and SM5–1 Fabs to pEW62, pEW21 and pEW2 was measured by biolayer interferometry.
(TIF)

S2 Fig Kinetic analysis of Fab binding to gB variants measured by BLI. gB variants were immobilized to biosensors and immersed in the indicated concentrations of serially diluted Fabs. Solid lines represent the raw data, and the dashed lines represent the theoretical fit. Data are representative of at least two measurements carried out on independent serial dilutions of Fabs. A summary of the kinetic data is provided in S2 Table. MLCB12 did not bind any constructs in Fab form and thus was excluded from the analysis.
(TIF)

S3 Fig Gating strategy on sorting pEW62 double positive single B cells. Human PBMCs from HCMV seropositive donors were thawed and B-cells enriched by negative selection. The cells were then stained with pEW62 conjugated to streptavidin-phycoerythrin (pEW62-PE) or streptavidin-allophycocyanin, and PY-gamma conjugated to streptavidin-phycoerythin-Dylite650, Gating as follows: Single Cells> Lymphocytes> CD3^-^, CD14^-^, Live/Dead^-^ > CD19^+^, IgG^+^> IgD^-^, IgG^+^> Decoy^-^ > pEW62^++^.
(TIF)

S4 Fig Permeabilized cell staining gating strategy and extended data. **(A)** Gating strategy to measure mAb binding to wildtype gB. 293-E6 cells were transiently transfected with gB from strain AD169 and permeabilized and incubated with the indicated antibodies, followed by a PE-conjugated anti-IgG secondary mAb and a viability stain. The bar gate in the right-hand panels indicates the percentage of cells that stain positive with the mAb (See S5 Fig). **(B-C)** The same data shown in Fig 4C, displayed as the mean fluorescence intensity of PE staining (MFI-PE) of live cells. The dashed line indicates the MFI of the isotype control.
(TIF)

S5 Fig Establishment of neutralization assay procedures and extended data. HCMV_GFP_ was tested at various MOIs and time points to determine the appropriate conditions for downstream neutralization assays. The virus was pre-incubated in media for 2 hours to mimic neutralization assay conditions, then added to confluent HFFs in a 96-well plate for the indicated times (as described in M&Ms). The plates were fixed with 4% PFA and read for bulk GFP signal (RFU). Fold change was calculated by dividing raw RFU values by RFUs of uninfected wells (background). This fold change was plotted above, with each point representing a technical replicate. The minimum MOI and time-point combination that provides a > 5-fold dynamic range was selected for downstream assays and is plotted in the corresponding strain color in Fig 5. TB40/e_GFP_ and TR_GFP_ do not reach the same titers as AD169_GFP,_ so the conditions were modified for those strains accordingly. **(A)** AD169_GFP_ was tested at 18 hrs. An MOI of 30 was selected. **(B-C)** TR_GFP_ was tested at 36 and 48 hours. TR and TB40/e_GFP_ reached similar titers, so TR_GFP_ was not tested at 18 hours, given the low RFU values exhibited by TB40/e_GFP_ at this time point. An MOI of 5 at 48 hours was selected to mitigate excess reagent usage. **(D-F)** TB40/e_GFP_ was tested at 18, 36, and 48 hours. An MOI of 5 at 48 hours was selected to mitigate excess reagent usage. **(G-I)** Percent neutralization of 12 newly isolated mAbs. Each mAb was tested at 100 μg/mL against (A) AD169_GFP_, (B) TR_GFP_, and (C) TB40/_eGFP_ in HFFs to determine neutralization potential for each strain. Known neutralizing antibodies 1G2 and SM5–1 were used as controls. Each data point is a biological replicate, which is an average of three technical replicates. The dashed line indicates our cutoff value of 60% neutralization. mAbs above this threshold were titrated. See S6 Fig for titration curves.
(TIF)

S6 Fig Titration curves for all the nAbs against three tested strains of HCMV. nAb and tested HCMV strain are indicated in the title of each graph. If a mAb did not neutralize a given strain, the titration curve is not included. The curves are a summary of n = 3 biological replicates, which were performed in technical triplicate. Antibody concentrations are in μg/mL.
(TIF)

S7 Fig MLCB1, MLCB3, and MLCB7 bind linear epitopes on HCMV gB. **(A)** Coomassie-stained SDS-PAGE gel of pEW62-HisAvi in reducing conditions. **(B)** pEW62-HisAvi was subjected to SDS-PAGE followed by Western blot analysis with the indicated mAbs. Neutralizing mAbs are indicated with an asterisk. N-terminal (N) and C-terminal (C) furin cleavage fragments are bounded by red and blue lines, respectively.
(TIF)

S8 Fig pEW62 was saturated with Fabs prior to NS-EM data collection. Size-exclusion chromatography (SEC) traces of pEW62/Fab complex preparations. Fab and pEW62 were mixed at a 2-fold molar excess of Fab to gB monomer (in 1X PBS buffer) to final volume of 250 µL and incubated on ice overnight. The samples were then run over a Superose 6 Increase 10/300 GL column. Each pEW62 + Fab SEC trace (color) is overlaid with a SEC trace for pEW62 alone (black). Colors are as in Fig 7. Positions of pEW62 alone, pEW62/Fab complex, and Fab alone are indicated on each trace.
(TIF)

S9 Fig Representative particles of each gB/Fab complex. Four representative particles for each Fab in complex with pEW62. A total of 30,000 – 50,000 particles were captured for each complex. Complexes were purified by size-exclusion (see S8 Fig), cross-linked, and stained with uranyl formate prior to data collection. Micrographs were captured at 49,000x magnification, and pictured particles were further enlarged for clarity. Particles are cropped to the same scale, as indicated by the scale bar beneath each set of images.
(TIF)

S10 Fig Sequence variation analysis of AD169, TR, and TB40/e pinpoints the approximate location of the MLCB9 epitope. (A) Surface model of the prefusion HCMV gB (*RCSB 7KDP*) with dI, dII, and dIV shown in blue, green, and orange, respectively. Beginning and end of unresolved dII sequence is shown in magenta. Unresolved dII region is represented by black dashed curved line. Putative MLCB9 epitope is labeled in black/teal. SM5–1 epitope is shown in salmon. 1G2 epitope is shown in yellow. Sites of sequence variations between AD169, TR, and TB40/e in dI and dII are shown in red. 27–156 binding domain (dIV, AD-1) is shown in orange. (B) Sequence alignment of AD169, TR, and TB40/e in domain I. Specific residues that differ are boxed in red. 1G2 epitope is boxed in yellow, as in (A). (C) Sequence alignment of AD169, TR, and TB40/e in domain II. Specific residues that differ are boxed in red. First and last residues in unresolved region are boxed in magenta, as in (A). Unresolved region, corresponding to black dashed line in (A), is labelled. SM5–1 epitope is boxed in salmon, as in (A). Residue in the putative MLCB9 epitope is boxed in teal. *Created in BioRender. McClave, M. (2025)* *https://BioRender.com/0bqcmxs*.
(TIF)

S11 Fig Transfected cell surface gating strategy and extended data. **(A)** Gating strategy to measure mAb binding to wildtype gB. 293-E6 cells were transiently transfected with gB from strain AD169 and incubated with the indicated antibodies, followed by a PE-conjugated anti-IgG secondary mAb and a viability stain. The bar gate in the right-hand panels indicates the percentage of cells that stain positive with the mAb. **(B-C)** The same data shown in Fig 8A, displayed as the mean fluorescence intensity of PE staining (MFI-PE) of live cells. The dashed line indicates the MFI of the isotype control. Negative values were given a value of 1 for graphical purposes in C.
(TIF)

S12 Fig Gating strategy to measure mAb binding to cells infected with HCMV. HFFs were infected with AD169_GFP_ at an MOI of 3 overnight. They were incubated with the indicated antibodies, followed by a PE-conjugated anti-IgG secondary mAb as described in the Materials and Methods. The bar gate in the right-hand panels indicates the percentage of cells that stain positive with the mAb.
(TIF)

S1 Table HCMV gB construct list. All constructs were made in a HCMV AD169 gB (“7M”: Y155G/I156H/Y157R/Y206H/W240A/L241T/Y242H) background. (First column from left) Name of HCMV gB mutant construct. Mutations added beyond the indicated background are indicated in columns labeled (Substitution Section 1) and (Substitution Section 2) in order of residue sequence number. Disulfides added to the construct are indicated separately in the (Disulfides) column. (Last residue of ectodomain) indicates the last numbered residue of HCMV gB used in the construct. No additional truncations were made unless explicitly indicated. (Linker) describes the covalent linker used between the last residue of the ectodomain and the (Trimerization Tag). Finally, [Conformations(s) (NS-EM)] describes the conformations of gB observed when the sample was viewed under negative-stain electron microscopy.
(XLSX)

S2 Table Kinetic Analysis of mAb binding to gB variants measured by BLI. Kinetic parameters of binding interactions between the indicated Fabs with the indicated gB variants as measured by BLI. The kinetic parameters for each gB variant and Fab are calculated and shown in the table. K_on_ (1/Ms) refers to the association rate constant which measures the speed of complex formation between the ligand and the analyte. K_dis_ (1/s) refers to the dissociation rate constant which measures the speed of the analyte breaking off from the ligand. KD (M) refers to the equilibrium dissociation constant calculated by dividing K_dis_ (1/s) by K_on_ (1/Ms). K_on_ Error and K_dis_ Error refer to the standard error of the mean calculated from their theoretical fitting curve values. Data shown is the average of at least two independent measurements on serial dilutions of Fabs. KD values are reported in Fig 6. Binding traces are shown in S2 Fig. N/A indicates no binding was observed, and the KD is reported as twice the highest Fab concentration tested.
(DOCX)

S3 Table Sequences of 12 new anti-HCMV gB antibodies. (Isolated antibody) indicates the name of each mAb. (Epitope Domain) indicates which structural domain the mAbs bind as defined by the experiments in Fig 6 and colored in accordance with the domain map in Fig 1. The heavy chain V-gene, CDRH3, and heavy chain GenBank accession number are indicated in their corresponding columns as are the light chain V-gene, CDRL3, and the light chain GenBank accession number.
(XLSX)
