# Supplementary figures and images for: Isolation and characterization of broadly-neutralizing anti-HCMV-gB antibodies from human donors using a prefusion-stabilized HCMV gB variant
Source: PLoS Pathog. 2026 Feb 5;22(2):e1013950. doi: 10.1371/journal.ppat.1013950 (PMC12890226; doi:10.1371/journal.ppat.1013950)

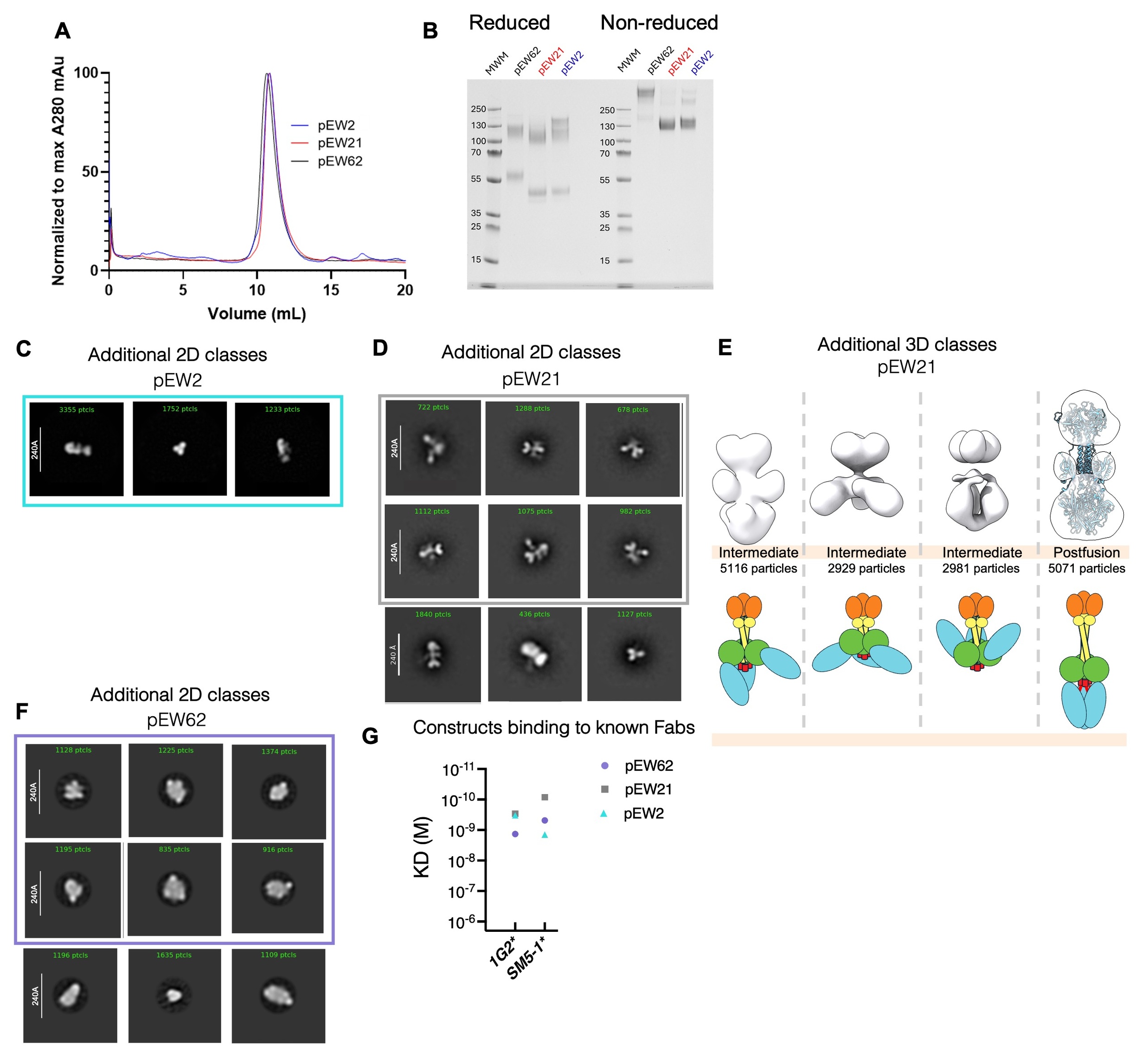

Supplement: S1 Fig — (A) purified pEW62, pEW21 and pEW2 were subjected to size exclusion chromatography on an Enrich650 column and the chromatograms are overlaid as indicated. (B) pEW62, pEW21 and pEW2 were separated by SDS-PAGE reducing and non-reducing conditions and then stained with Coomassie blue. MWM = molecular weight marker. (C-F) Additional 2D and 3D classes of gB constructs. Each set of 2D classes for each construct is taken from the same 2D classification “job” in CryoSPARC and includes particles used in generating ab-initio reconstructions (see M&Ms for detailed processing strategy). Trash particles are not shown. Scale for each row of images is indicated on the left-hand side of the first image. (C) All particles are classified as postfusion. (D) Particles classified as intermediate are boxed in gray, corresponding to coloring in Fig 2E and 2F. Postfusion particles are unboxed. These particles have different dimensions than pre- or postfusion, they are much shorter. (E) Additional 3D classes of pEW21 with a cartoon schematic of the hypothesized conformations they represent. From left to right, particle numbers comprising each class are as follows: 5116, 2929, 2981, and 5071. In the case of the first class, it is an average of multiple intermediate particles that have one or more dI “arms” swinging upwards. Flexible regions of particles are not included in averages, as they are more challenging to capture. Thus, the ends of dI, which vary between particles, are not in the average. The same explanation can be used for the other classes. (F) Particles classified as “prefusion” are boxed in purple, corresponding to coloring in Fig 2H and 2I. Postfusion particles are unboxed. (G) The affinity of 1G2 and SM5–1 Fabs to pEW62, pEW21 and pEW2 was measured by biolayer interferometry. (TIF) [file ppat.1013950.s001.tif]

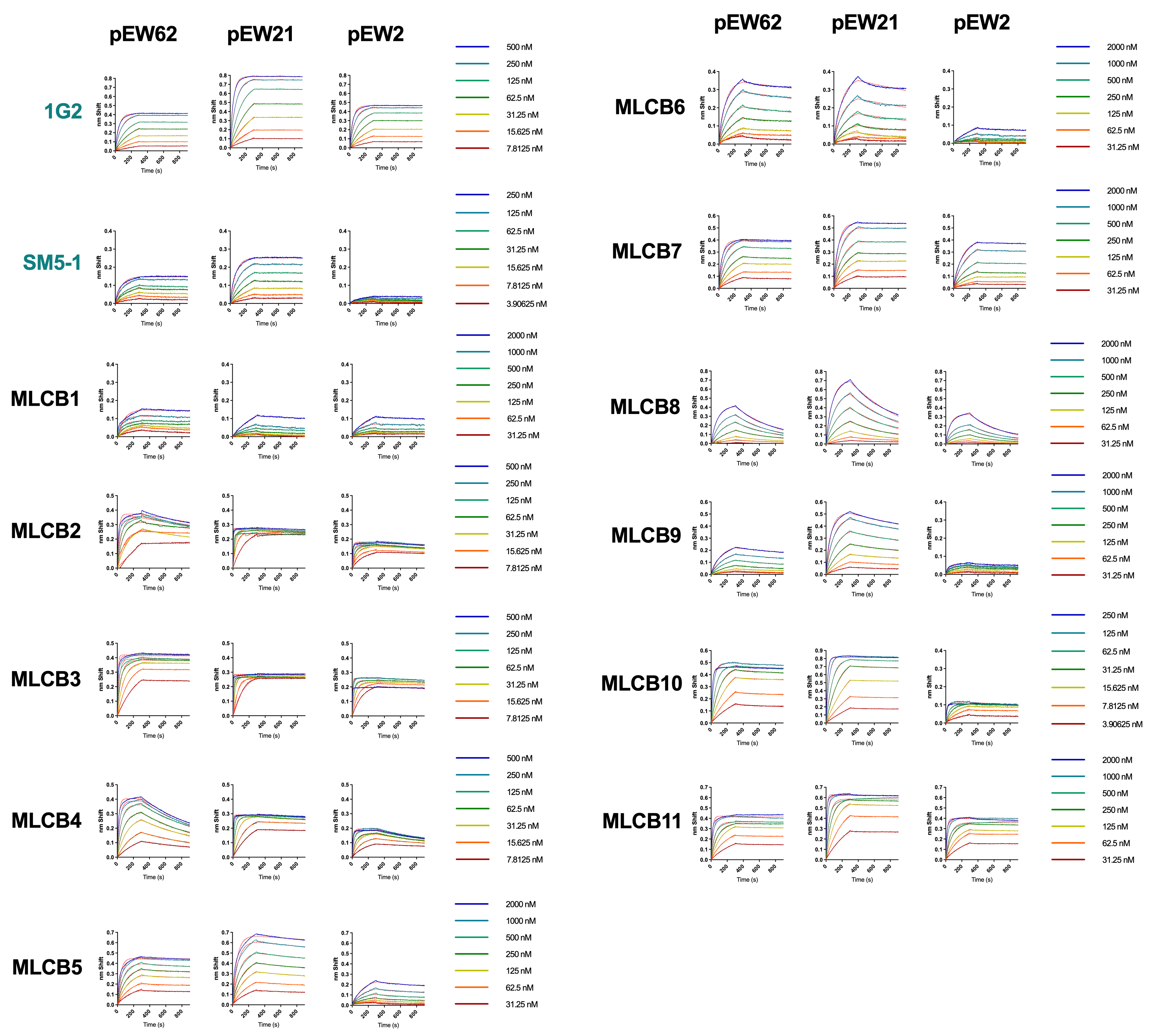

Supplement: S2 Fig — gB variants were immobilized to biosensors and immersed in the indicated concentrations of serially diluted Fabs. Solid lines represent the raw data, and the dashed lines represent the theoretical fit. Data are representative of at least two measurements carried out on independent serial dilutions of Fabs. A summary of the kinetic data is provided in S2 Table. MLCB12 did not bind any constructs in Fab form and thus was excluded from the analysis. (TIF) [file ppat.1013950.s002.tif]

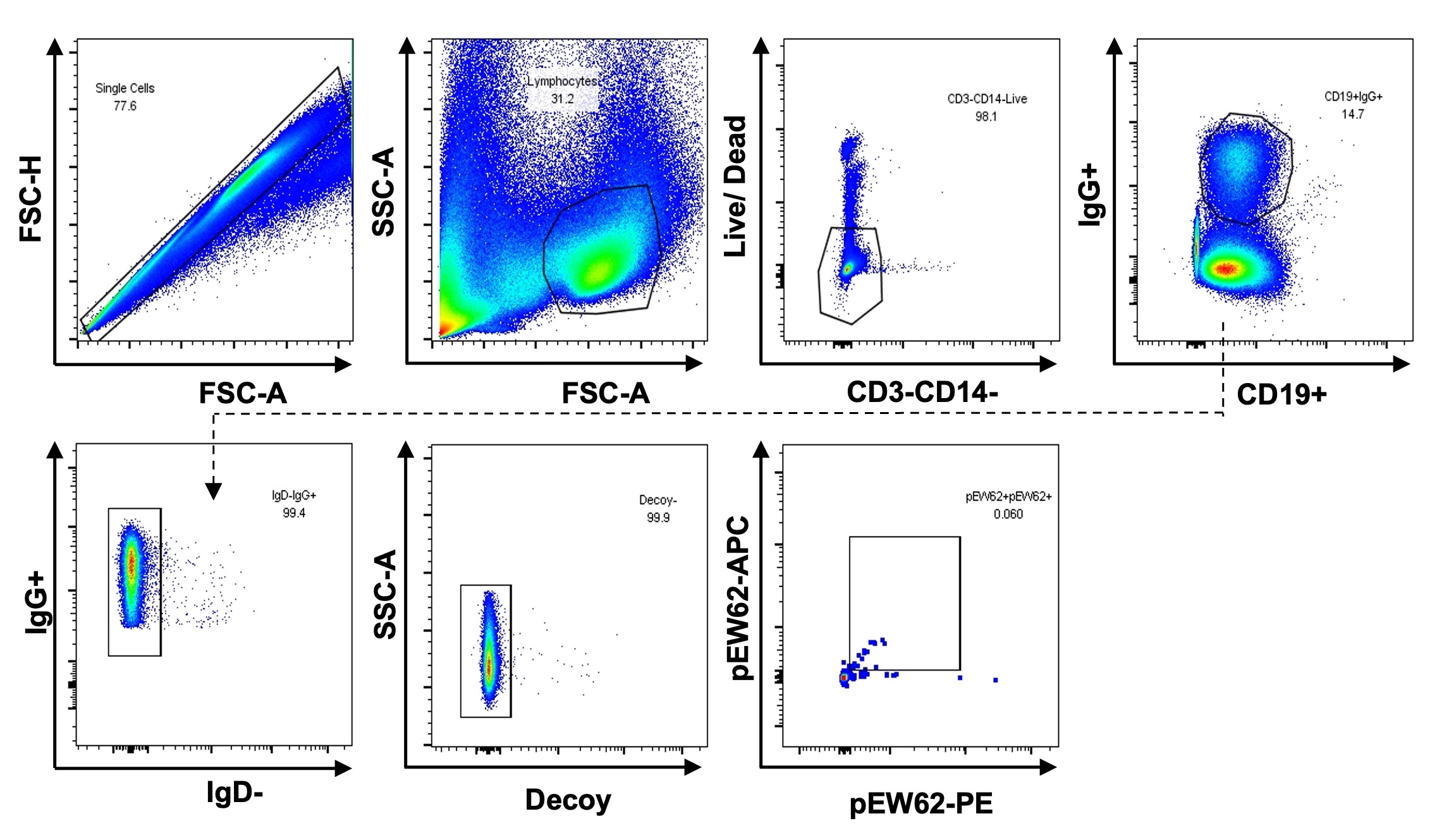

Supplement: S3 Fig — Human PBMCs from HCMV seropositive donors were thawed and B-cells enriched by negative selection. The cells were then stained with pEW62 conjugated to streptavidin-phycoerythrin (pEW62-PE) or streptavidin-allophycocyanin, and PY-gamma conjugated to streptavidin-phycoerythin-Dylite650, Gating as follows: Single Cells> Lymphocytes> CD3-, CD14-, Live/Dead- > CD19+, IgG+> IgD-, IgG+> Decoy- > pEW62++. (TIF) [file ppat.1013950.s003.tif]

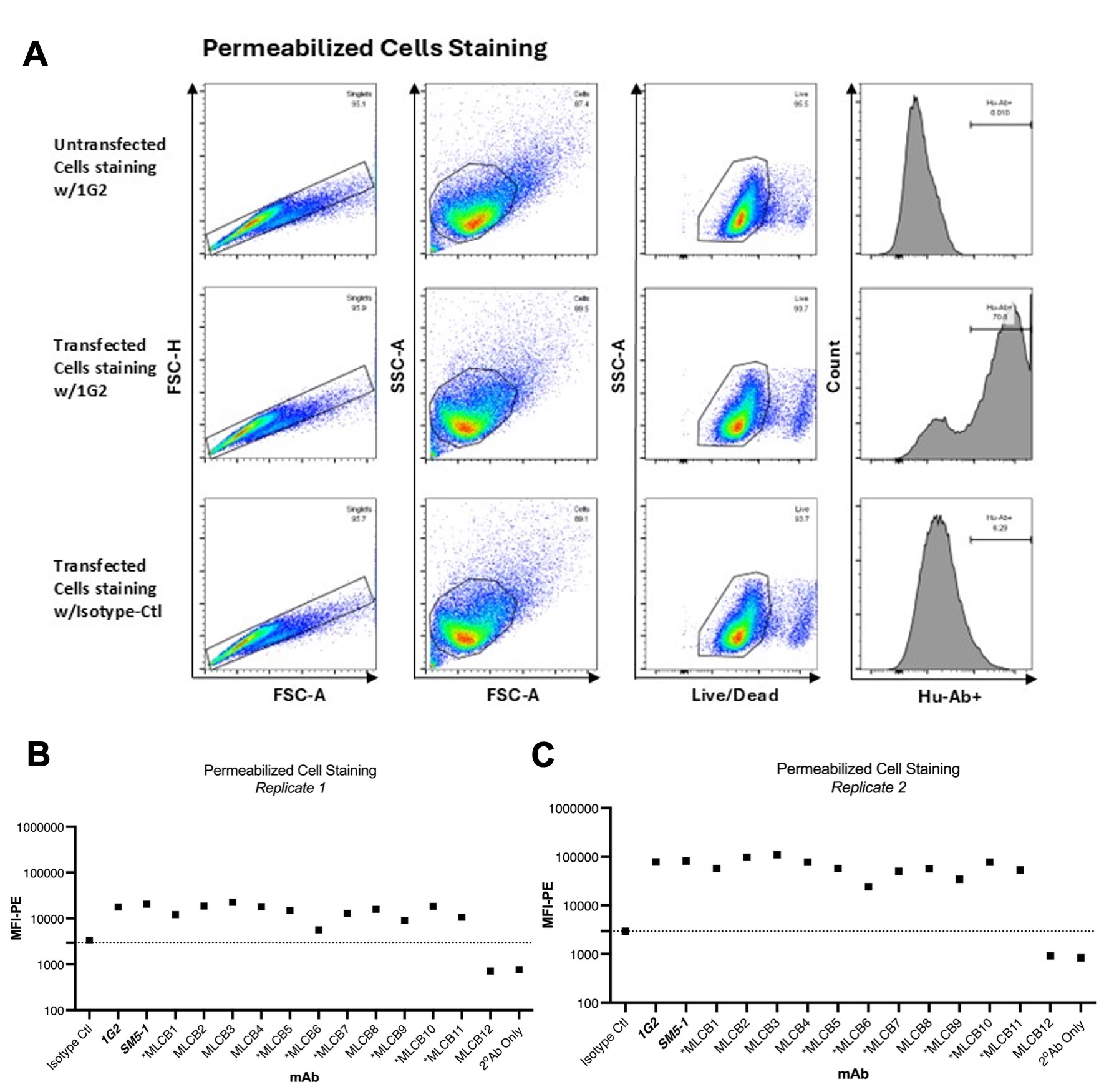

Supplement: S4 Fig — (A) Gating strategy to measure mAb binding to wildtype gB. 293-E6 cells were transiently transfected with gB from strain AD169 and permeabilized and incubated with the indicated antibodies, followed by a PE-conjugated anti-IgG secondary mAb and a viability stain. The bar gate in the right-hand panels indicates the percentage of cells that stain positive with the mAb (See S5 Fig). (B-C) The same data shown in Fig 4C, displayed as the mean fluorescence intensity of PE staining (MFI-PE) of live cells. The dashed line indicates the MFI of the isotype control. (TIF) [file ppat.1013950.s004.tif]

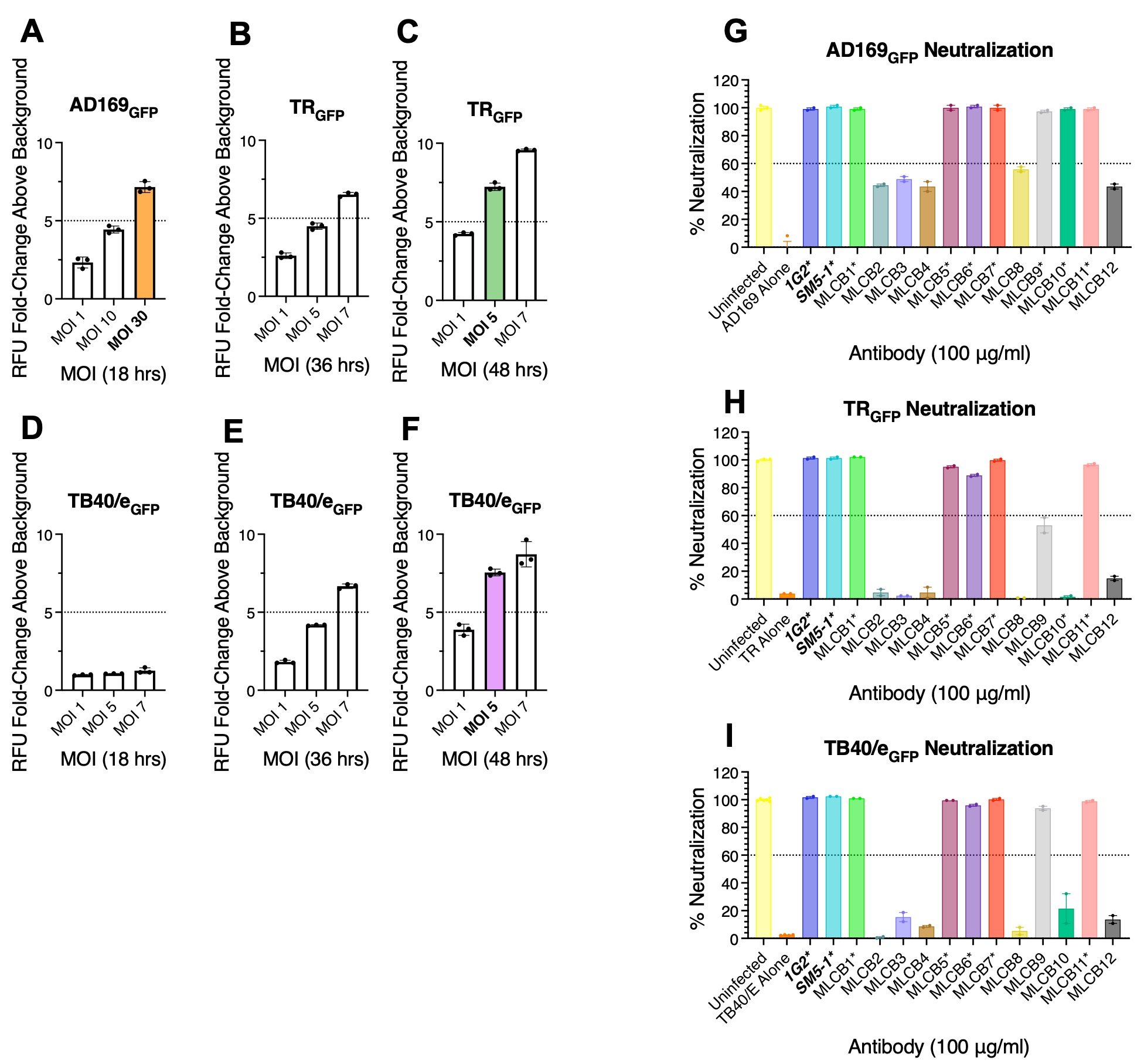

Supplement: S5 Fig — HCMVGFP was tested at various MOIs and time points to determine the appropriate conditions for downstream neutralization assays. The virus was pre-incubated in media for 2 hours to mimic neutralization assay conditions, then added to confluent HFFs in a 96-well plate for the indicated times (as described in M&Ms). The plates were fixed with 4% PFA and read for bulk GFP signal (RFU). Fold change was calculated by dividing raw RFU values by RFUs of uninfected wells (background). This fold change was plotted above, with each point representing a technical replicate. The minimum MOI and time-point combination that provides a > 5-fold dynamic range was selected for downstream assays and is plotted in the corresponding strain color in Fig 5. TB40/eGFP and TRGFP do not reach the same titers as AD169GFP, so the conditions were modified for those strains accordingly. (A) AD169GFP was tested at 18 hrs. An MOI of 30 was selected. (B-C) TRGFP was tested at 36 and 48 hours. TR and TB40/eGFP reached similar titers, so TRGFP was not tested at 18 hours, given the low RFU values exhibited by TB40/eGFP at this time point. An MOI of 5 at 48 hours was selected to mitigate excess reagent usage. (D-F) TB40/eGFP was tested at 18, 36, and 48 hours. An MOI of 5 at 48 hours was selected to mitigate excess reagent usage. (G-I) Percent neutralization of 12 newly isolated mAbs. Each mAb was tested at 100 μg/mL against (A) AD169GFP, (B) TRGFP, and (C) TB40/eGFP in HFFs to determine neutralization potential for each strain. Known neutralizing antibodies 1G2 and SM5–1 were used as controls. Each data point is a biological replicate, which is an average of three technical replicates. The dashed line indicates our cutoff value of 60% neutralization. mAbs above this threshold were titrated. See S6 Fig for titration curves. (TIF) [file ppat.1013950.s005.tif]

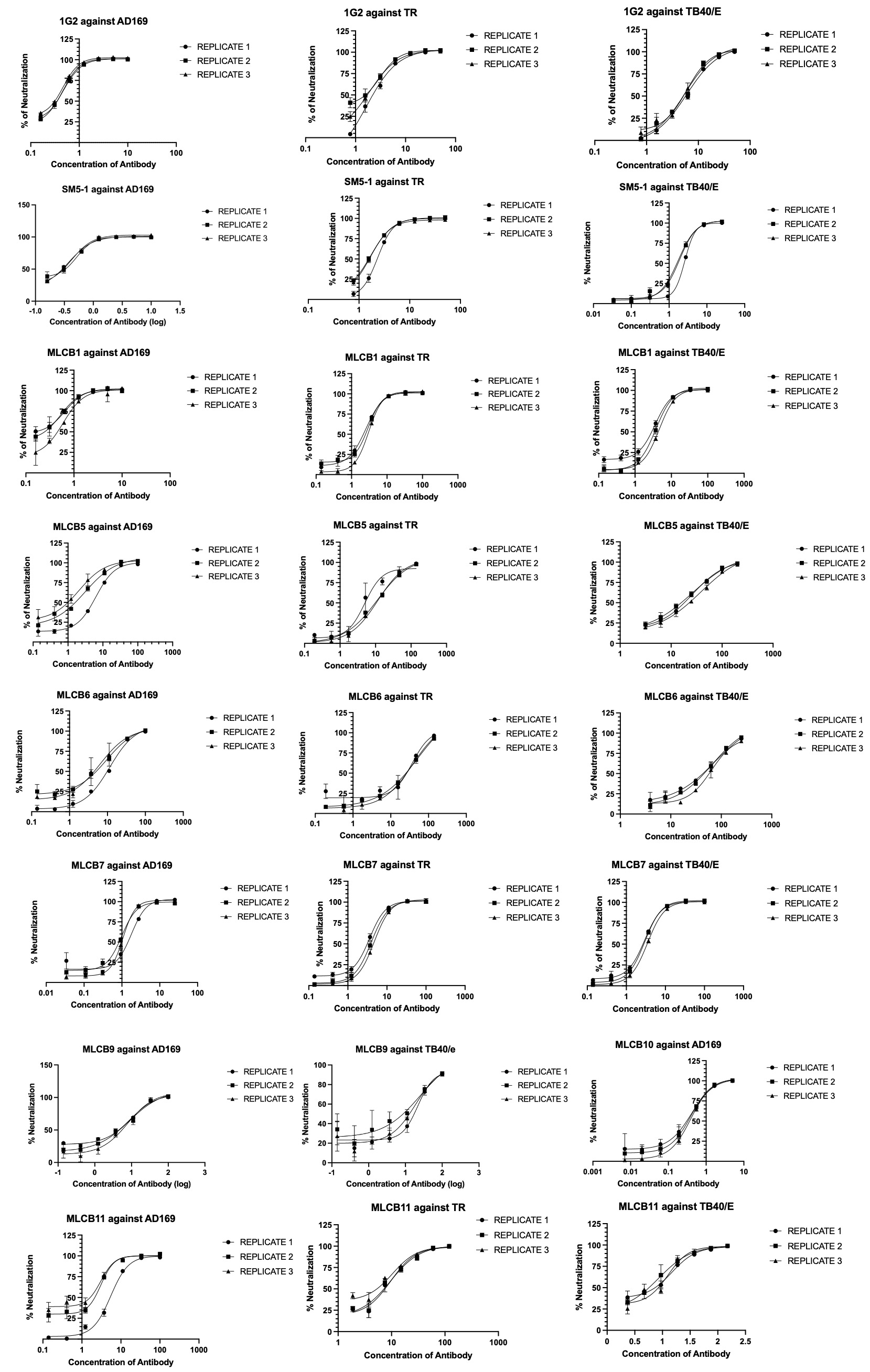

Supplement: S6 Fig — If a mAb did not neutralize a given strain, the titration curve is not included. The curves are a summary of n = 3 biological replicates, which were performed in technical triplicate. Antibody concentrations are in μg/mL. (TIF) [file ppat.1013950.s006.tif]

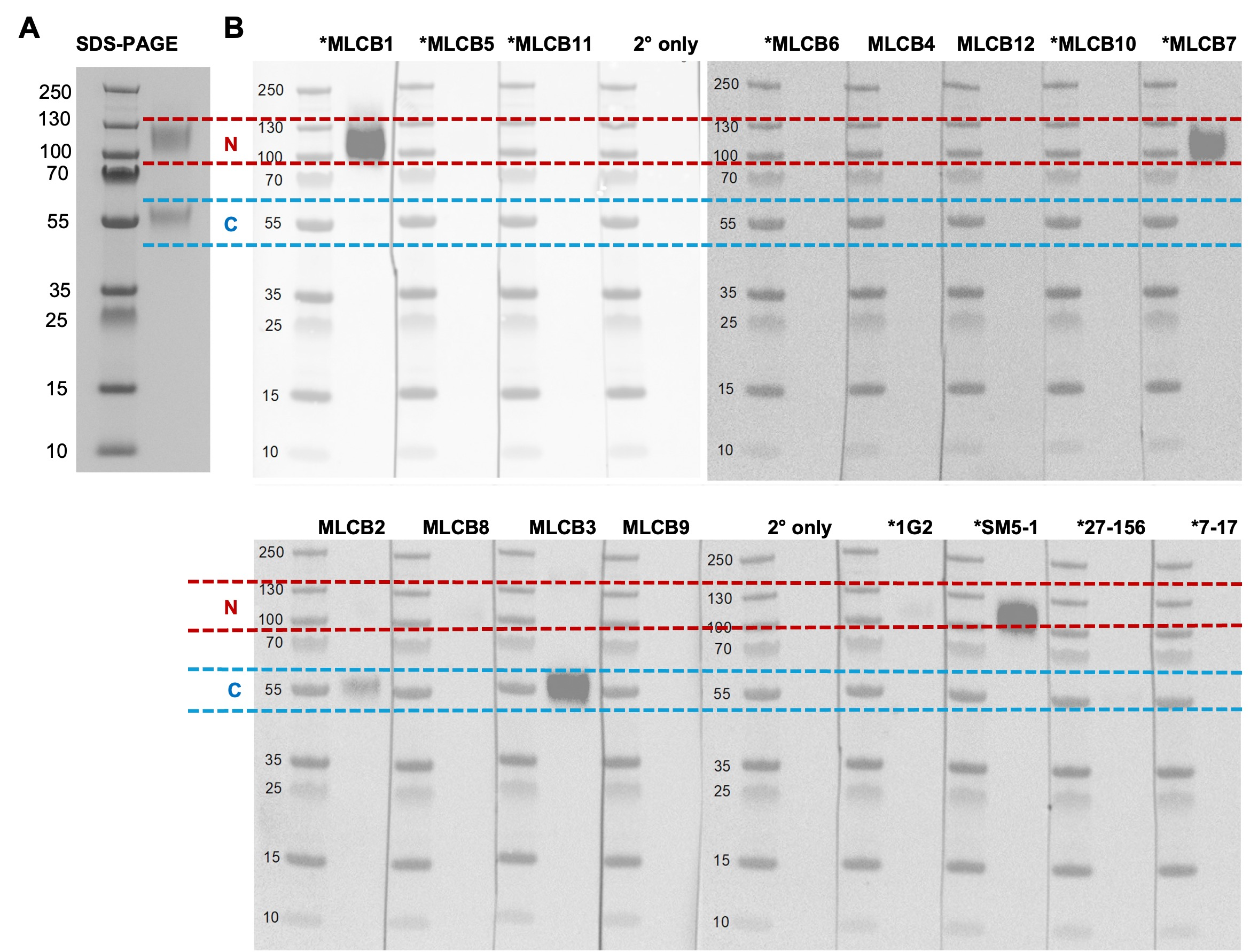

Supplement: S7 Fig — (A) Coomassie-stained SDS-PAGE gel of pEW62-HisAvi in reducing conditions. (B) pEW62-HisAvi was subjected to SDS-PAGE followed by Western blot analysis with the indicated mAbs. Neutralizing mAbs are indicated with an asterisk. N-terminal (N) and C-terminal (C) furin cleavage fragments are bounded by red and blue lines, respectively. (TIF) [file ppat.1013950.s007.tif]

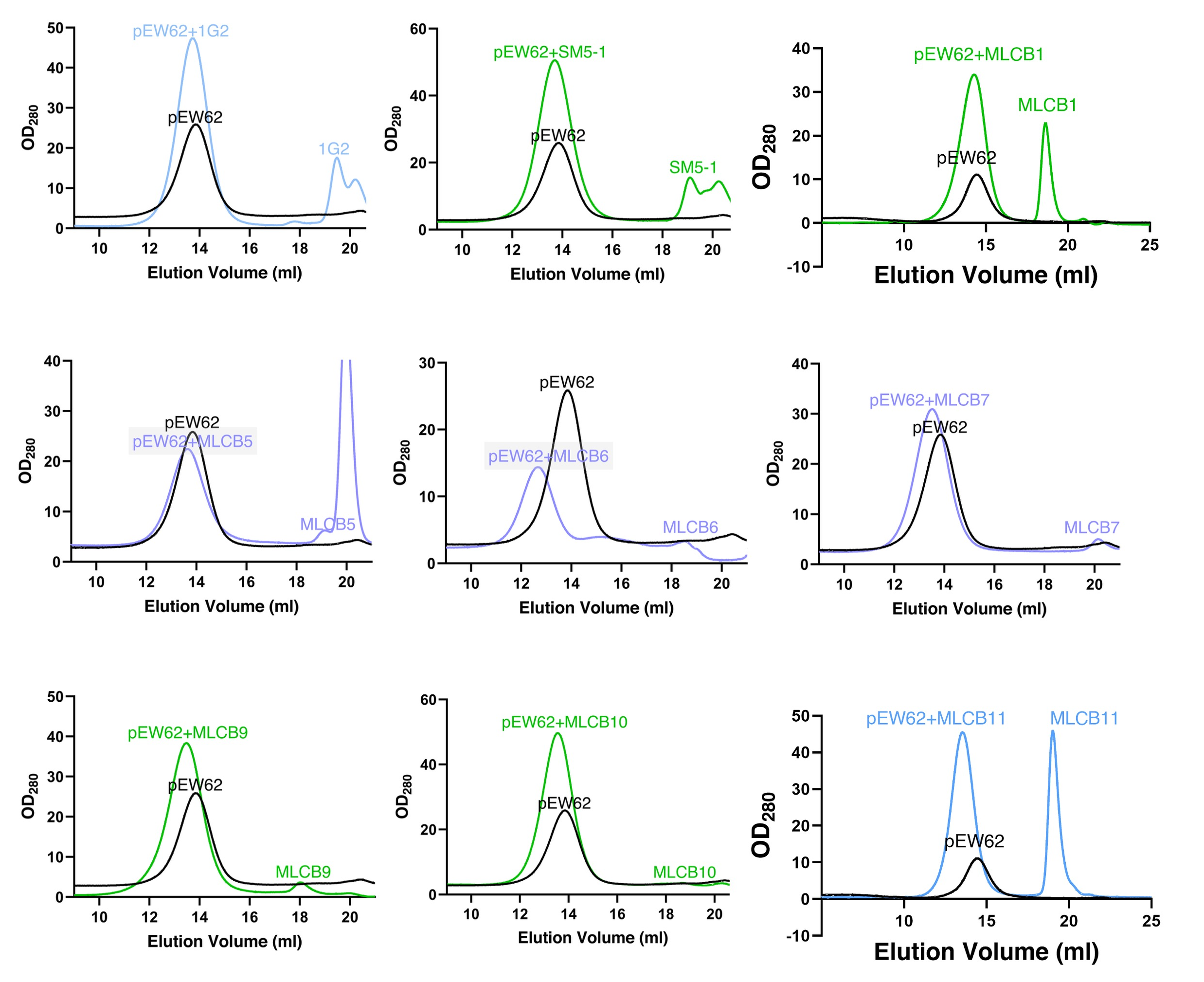

Supplement: S8 Fig — Size-exclusion chromatography (SEC) traces of pEW62/Fab complex preparations. Fab and pEW62 were mixed at a 2-fold molar excess of Fab to gB monomer (in 1X PBS buffer) to final volume of 250 µL and incubated on ice overnight. The samples were then run over a Superose 6 Increase 10/300 GL column. Each pEW62 + Fab SEC trace (color) is overlaid with a SEC trace for pEW62 alone (black). Colors are as in Fig 7. Positions of pEW62 alone, pEW62/Fab complex, and Fab alone are indicated on each trace. (TIF) [file ppat.1013950.s008.tif]

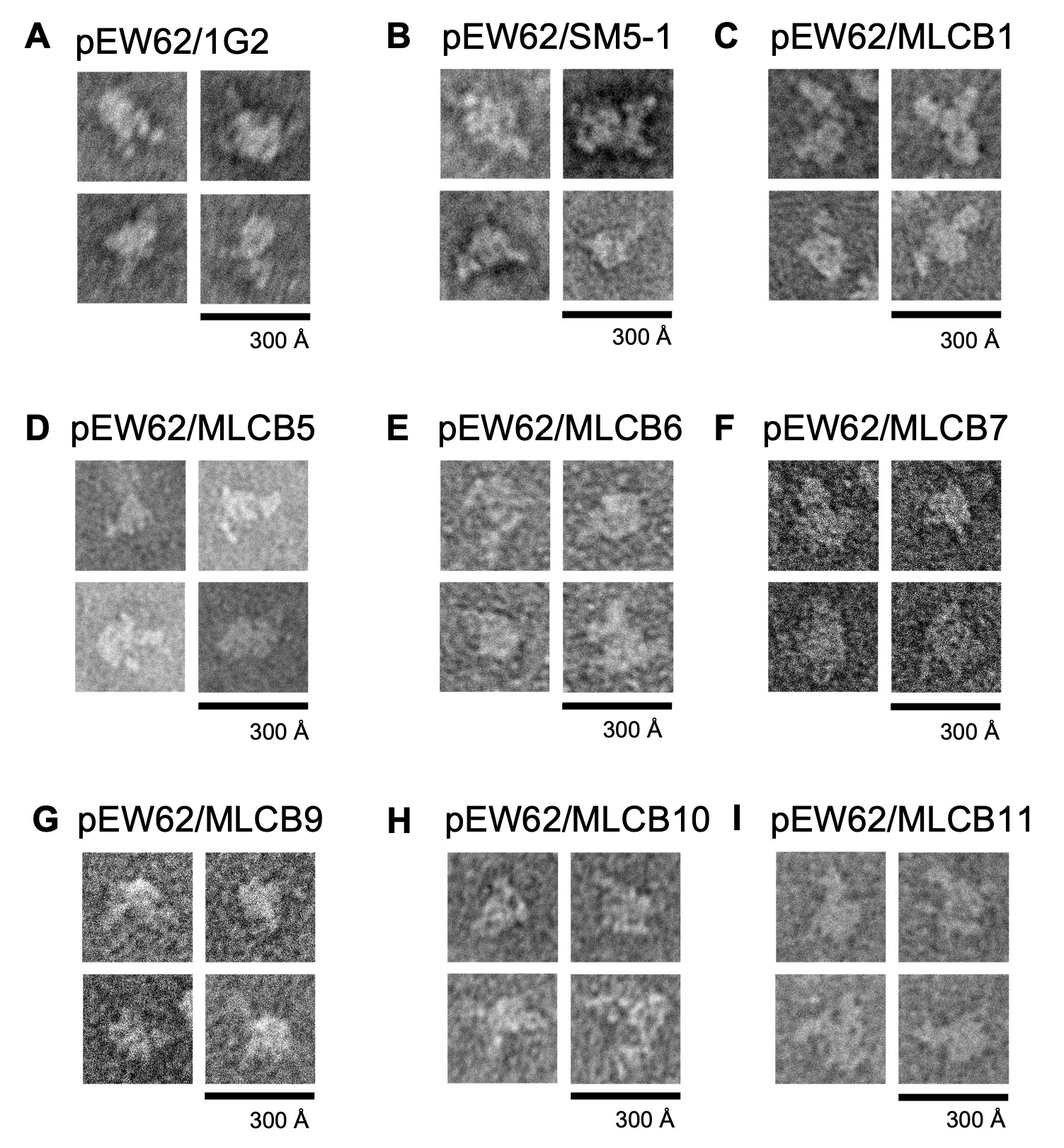

Supplement: S9 Fig — Four representative particles for each Fab in complex with pEW62. A total of 30,000 – 50,000 particles were captured for each complex. Complexes were purified by size-exclusion (see S8 Fig), cross-linked, and stained with uranyl formate prior to data collection. Micrographs were captured at 49,000x magnification, and pictured particles were further enlarged for clarity. Particles are cropped to the same scale, as indicated by the scale bar beneath each set of images. (TIF) [file ppat.1013950.s009.tif]

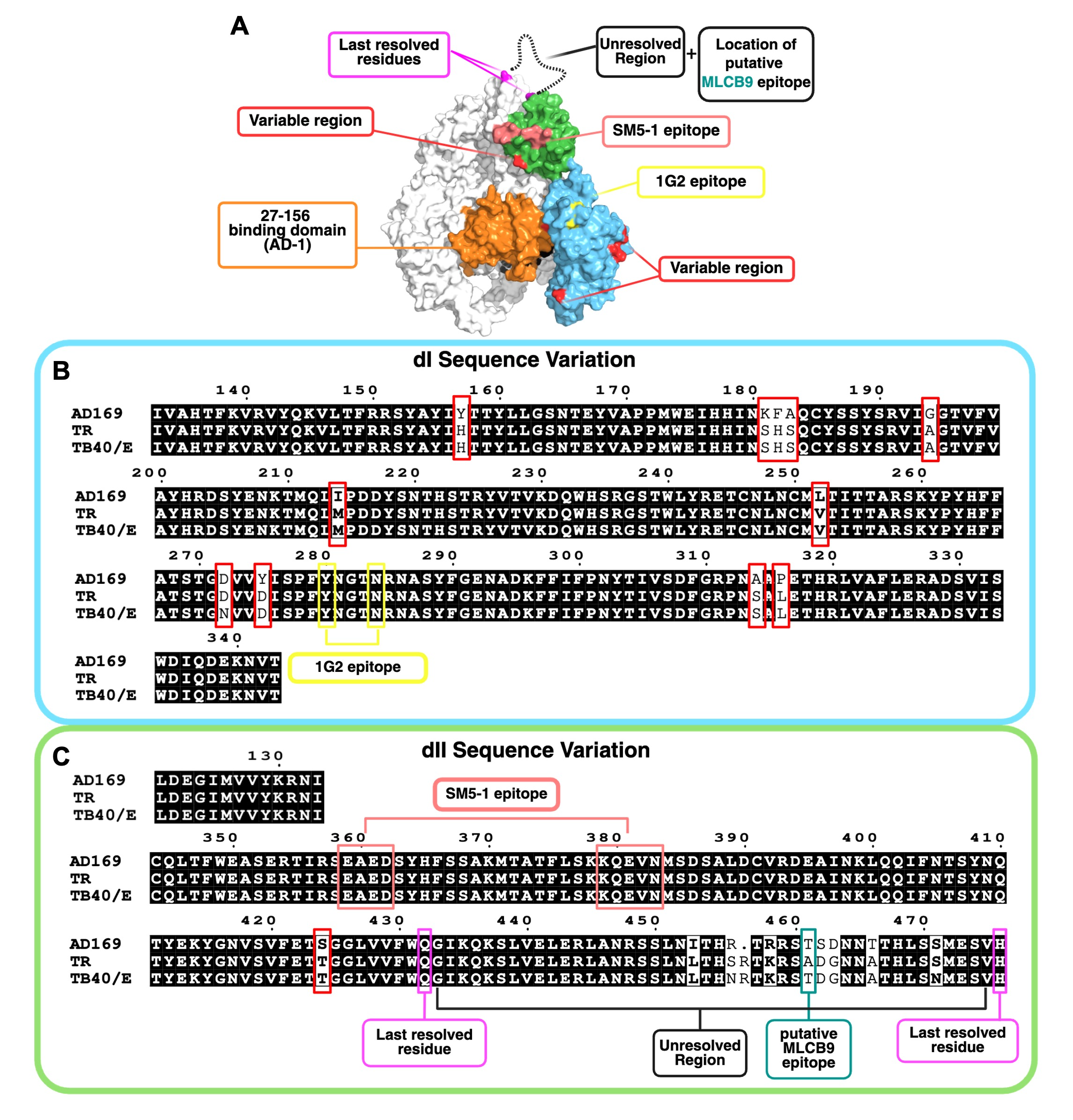

Supplement: S10 Fig — (A) Surface model of the prefusion HCMV gB (RCSB 7KDP) with dI, dII, and dIV shown in blue, green, and orange, respectively. Beginning and end of unresolved dII sequence is shown in magenta. Unresolved dII region is represented by black dashed curved line. Putative MLCB9 epitope is labeled in black/teal. SM5–1 epitope is shown in salmon. 1G2 epitope is shown in yellow. Sites of sequence variations between AD169, TR, and TB40/e in dI and dII are shown in red. 27–156 binding domain (dIV, AD-1) is shown in orange. (B) Sequence alignment of AD169, TR, and TB40/e in domain I. Specific residues that differ are boxed in red. 1G2 epitope is boxed in yellow, as in (A). (C) Sequence alignment of AD169, TR, and TB40/e in domain II. Specific residues that differ are boxed in red. First and last residues in unresolved region are boxed in magenta, as in (A). Unresolved region, corresponding to black dashed line in (A), is labelled. SM5–1 epitope is boxed in salmon, as in (A). Residue in the putative MLCB9 epitope is boxed in teal. Created in BioRender. McClave, M. (2025) https://BioRender.com/0bqcmxs. (TIF) [file ppat.1013950.s010.tif]

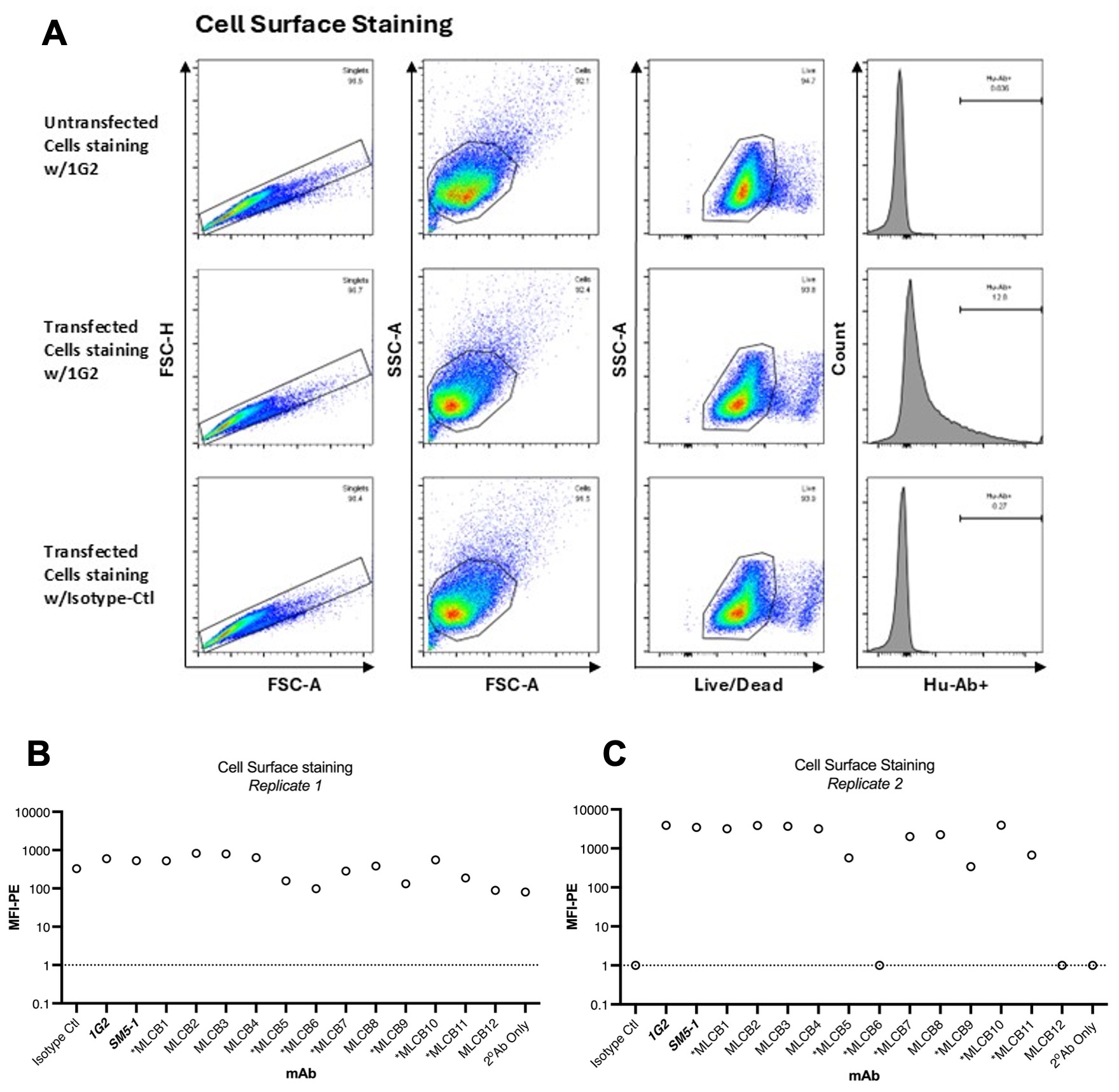

Supplement: S11 Fig — (A) Gating strategy to measure mAb binding to wildtype gB. 293-E6 cells were transiently transfected with gB from strain AD169 and incubated with the indicated antibodies, followed by a PE-conjugated anti-IgG secondary mAb and a viability stain. The bar gate in the right-hand panels indicates the percentage of cells that stain positive with the mAb. (B-C) The same data shown in Fig 8A, displayed as the mean fluorescence intensity of PE staining (MFI-PE) of live cells. The dashed line indicates the MFI of the isotype control. Negative values were given a value of 1 for graphical purposes in C. (TIF) [file ppat.1013950.s011.tif]

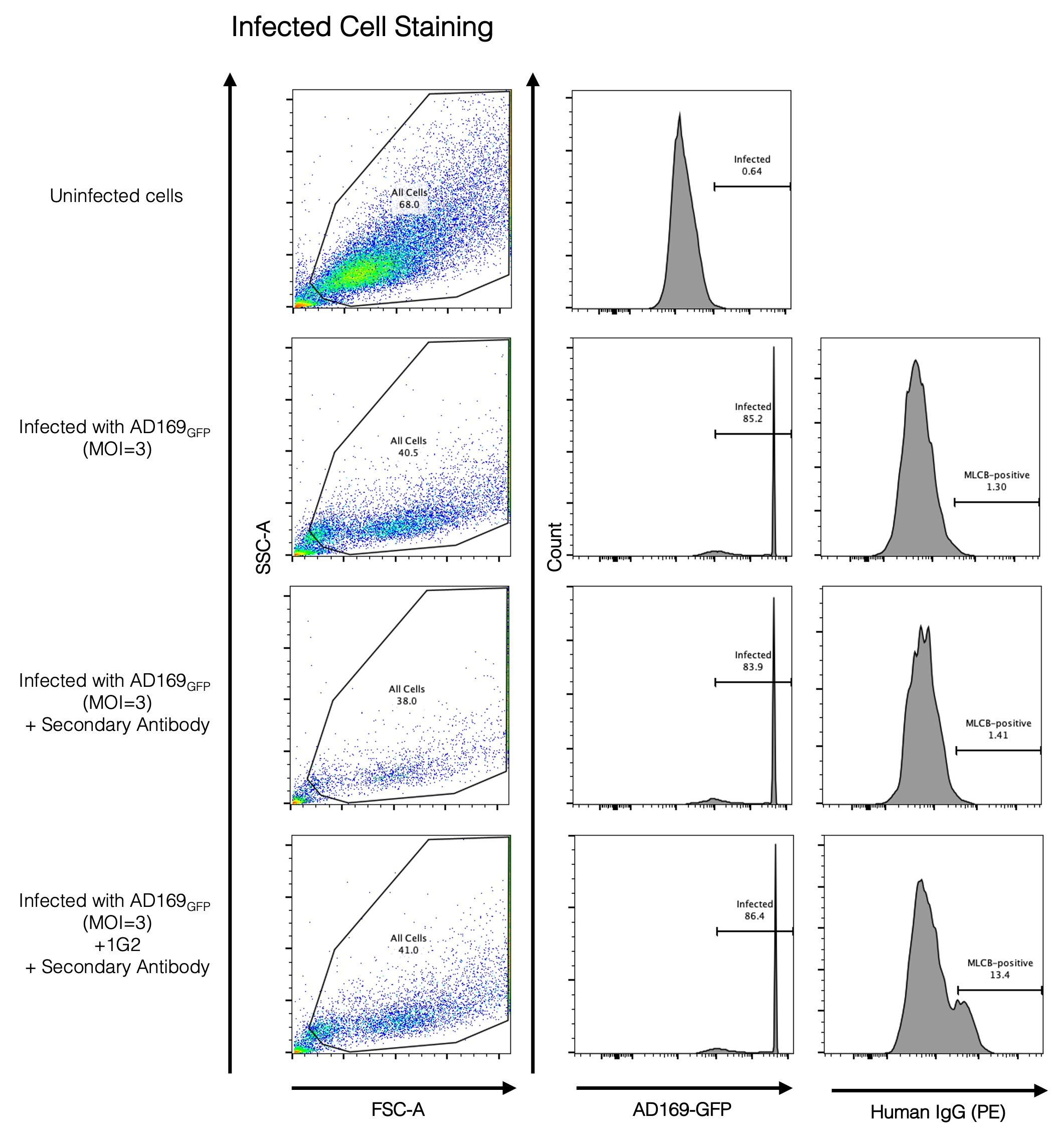

Supplement: S12 Fig — HFFs were infected with AD169GFP at an MOI of 3 overnight. They were incubated with the indicated antibodies, followed by a PE-conjugated anti-IgG secondary mAb as described in the Materials and Methods. The bar gate in the right-hand panels indicates the percentage of cells that stain positive with the mAb. (TIF) [file ppat.1013950.s012.tif]
