# Supplementary material for: Isolation and characterization of broadly-neutralizing anti-HCMV-gB antibodies from human donors using a prefusion-stabilized HCMV gB variant
Source: PLoS Pathog. 2026 Feb 5;22(2):e1013950. doi: 10.1371/journal.ppat.1013950 (PMC12890226; doi:10.1371/journal.ppat.1013950)
Supplement: S2 Table — Kinetic parameters of binding interactions between the indicated Fabs with the indicated gB variants as measured by BLI. The kinetic parameters for each gB variant and Fab are calculated and shown in the table. Kon (1/Ms) refers to the association rate constant which measures the speed of complex formation between the ligand and the analyte. Kdis (1/s) refers to the dissociation rate constant which measures the speed of the analyte breaking off from the ligand. KD (M) refers to the equilibrium dissociation constant calculated by dividing Kdis (1/s) by Kon (1/Ms). Kon Error and Kdis Error refer to the standard error of the mean calculated from their theoretical fitting curve values. Data shown is the average of at least two independent measurements on serial dilutions of Fabs. KD values are reported in Fig 6. Binding traces are shown in S2 Fig. N/A indicates no binding was observed, and the KD is reported as twice the highest Fab concentration tested. (DOCX) [file ppat.1013950.s014.docx]

**S2 Table. Kinetic Analysis of mAb binding to gB variants measured by BLI.**

| Fab (analyte) | gB variant  (ligand) | ***K_D_* (M)** | *k_on_*(1/Ms) | *k_on_* Error | *k_dis_*(1/s) | *k_dis_* Error |
| --- | --- | --- | --- | --- | --- | --- |
| SM51 | pEW62 | **4.85E-10** | 2.14E+05 | 2.93E+03 | 1.04E-04 | 5.57E-06 |
|  | pEW21 | **8.39E-11** | 2.13E+05 | 4.43E+03 | 1.79E-05 | 4.37E-06 |
|  | pEW2 | **1.41E-09** | 3.28E+04 | 6.29E+02 | 4.61E-05 | 9.75E-06 |
| 1G2 | pEW62 | **1.36E-09** | 7.63E+04 | 1.54E+03 | 1.11E-04 | 4.25E-06 |
|  | pEW21 | **2.90E-10** | 1.89E+05 | 3.74E+03 | 5.46E-05 | 3.45E-06 |
|  | pEW2 | **3.31E-10** | 2.19E+05 | 4.55E+03 | 7.11E-05 | 4.49E-06 |
| MLCB1 | pEW62 | **6.59E-09** | 4.97E+04 | 8.75E+02 | 3.27E-04 | 7.82E-06 |
|  | pEW21 | **7.12E-08** | 6.26E+03 | 1.38E+02 | 4.46E-04 | 8.07E-06 |
|  | pEW2 | **5.11E-08** | 3.23E+03 | 4.65E+01 | 1.65E-04 | 6.54E-06 |
| MLCB2 | pEW62 | **1.53E-09** | 1.99E+05 | 1.46E+03 | 3.04E-04 | 4.09E-06 |
|  | pEW21 | **1.00E-10** | 8.54E+05 | 5.04E+03 | 8.54E-05 | 1.80E-06 |
|  | pEW2 | **2.61E-10** | 6.57E+05 | 5.50E+03 | 1.71E-04 | 4.47E-06 |
| MLCB3 | pEW62 | **5.77E-11** | 4.53E+05 | 1.97E+03 | 2.61E-05 | 1.50E-06 |
|  | pEW21 | **1.63E-11** | 1.02E+06 | 6.13E+03 | 1.66E-05 | 2.46E-06 |
|  | pEW2 | **9.52E-11** | 7.77E+05 | 3.99E+03 | 7.40E-05 | 2.04E-06 |
| MLCB4 | pEW62 | **8.02E-09** | 1.40E+05 | 1.67E+03 | 9.11E-04 | 5.93E-06 |
|  | pEW21 | **2.94E-10** | 4.59E+05 | 1.98E+03 | 1.35E-04 | 1.74E-06 |
|  | pEW2 | **1.98E-09** | 3.45E+05 | 3.31E+03 | 6.25E-04 | 4.51E-06 |
| MLCB5 | pEW62 | **1.57E-09** | 6.94E+04 | 8.16E+02 | 1.08E-04 | 4.10E-06 |
|  | pEW21 | **3.71E-09** | 5.60E+04 | 6.38E+02 | 2.06E-04 | 3.62E-06 |
|  | pEW2 | **6.88E-08** | 5.44E+03 | 6.90E+01 | 3.75E-04 | 6.12E-06 |
| MLCB6 | pEW62 | **9.02E-09** | 2.76E+04 | 5.01E+02 | 2.49E-04 | 5.35E-06 |
|  | pEW21 | **3.10E-08** | 1.30E+04 | 2.18E+02 | 4.04E-04 | 7.36E-06 |
|  | pEW2 | **1.16E-07** | 2.20E+03 | 3.97E+01 | 2.56E-04 | 6.30E-06 |
| MLCB7 | pEW62 | **1.57E-09** | 5.90E+04 | 1.22E+03 | 8.91E-05 | 3.84E-06 |
|  | pEW21 | **3.41E-10** | 1.02E+05 | 1.58E+03 | 3.70E-05 | 4.18E-06 |
|  | pEW2 | **1.99E-09** | 2.58E+04 | 5.39E+02 | 5.15E-05 | 4.63E-06 |
| MLCB8 | pEW62 | **6.37E-08** | 2.61E+04 | 4.99E+02 | 1.66E-03 | 5.85E-06 |
|  | pEW21 | **1.67E-08** | 7.84E+04 | 3.85E+03 | 1.31E-03 | 6.67E-06 |
|  | pEW2 | **1.87E-08** | 1.23E+05 | 3.30E+03 | 2.30E-03 | 1.12E-05 |
| MLCB9 | pEW62 | **4.83E-08** | 1.09E+04 | 3.03E+02 | 5.24E-04 | 6.16E-06 |
|  | pEW21 | **1.43E-08** | 2.34E+04 | 3.41E+02 | 3.16E-04 | 3.10E-06 |
|  | pEW2 | **>2.00E-06** | N/A | N/A | N/A | N/A |
| MLCB10 | pEW62 | **2.84E-10** | 3.04E+05 | 2.01E+03 | 8.63E-05 | 1.70E-06 |
|  | pEW21 | **2.12E-10** | 2.34E+05 | 1.28E+03 | 4.96E-05 | 1.17E-06 |
|  | pEW2 | **2.65E-10** | 4.51E+05 | 4.07E+03 | 1.18E-04 | 3.49E-06 |
| MLCB11 | pEW62 | **7.01E-10** | 7.72E+04 | 6.04E+02 | 5.38E-05 | 2.46E-06 |
|  | pEW21 | **3.79E-10** | 7.54E+04 | 3.76E+02 | 2.82E-05 | 1.54E-06 |
|  | pEW2 | **5.14E-10** | 7.00E+04 | 5.13E+02 | 3.60E-05 | 2.31E-06 |
| MLCB12 | pEW62 | **>2.00E-06** | N/A | N/A | N/A | N/A |
|  | pEW21 | **>2.00E-06** | N/A | N/A | N/A | N/A |
|  | pEW2 | **>2.00E-06** | N/A | N/A | N/A | N/A |
